# Application of Microextraction-Based Techniques for Screening-Controlled Drugs in Forensic Context—A Review

**DOI:** 10.3390/molecules26082168

**Published:** 2021-04-09

**Authors:** Samir M. Ahmad, Oriana C. Gonçalves, Mariana N. Oliveira, Nuno R. Neng, José M. F. Nogueira

**Affiliations:** 1 Centro de Química Estrutural, Faculdade de Ciências, Universidade de Lisboa, 1749-016 Lisboa, Portugal; ocgp98@gmail.com (O.C.G.); mariananetoliveira@hotmail.com (M.N.O.); 2 Molecular Pathology and Forensic Biochemistry Laboratory, CiiEM, Campus Universitário—Quinta da Granja, Monte da Caparica, 2829-511 Caparica, Portugal; 3 Forensic and Psychological Sciences Laboratory Egas Moniz, Campus Universitário—Quinta da Granja, Monte da Caparica, 2829-511 Caparica, Portugal; 4 Departamento de Química e Bioquímica, Faculdade de Ciências, Universidade de Lisboa, 1749-016 Lisboa, Portugal

**Keywords:** microextraction-based techniques, screening methods, controlled drugs, opioids, cannabis, amphetamines, hallucinogens, dissociative drugs, new psychoactive substances, forensic matrices

## Abstract

The analysis of controlled drugs in forensic matrices, i.e., urine, blood, plasma, saliva, and hair, is one of the current hot topics in the clinical and toxicological context. The use of microextraction-based approaches has gained considerable notoriety, mainly due to the great simplicity, cost-benefit, and environmental sustainability. For this reason, the application of these innovative techniques has become more relevant than ever in programs for monitoring priority substances such as the main illicit drugs, e.g., opioids, stimulants, cannabinoids, hallucinogens, dissociative drugs, and related compounds. The present contribution aims to make a comprehensive review on the state-of-the art advantages and future trends on the application of microextraction-based techniques for screening-controlled drugs in the forensic context.

## 1. Introduction

Abuse and drug addiction, as well as their consequences, are one of the major issues in modern societies. The European Monitoring Center for Drugs and Drug Addiction (EMCDDA) classifies drugs as all substances that people take to achieve a change of mental, physical, or emotional state (i.e., psychoactive substances). This definition also includes tobacco, alcohol, medicinal drugs, and volatile substances (“inhalants”). On the other hand, controlled drugs are only those that are listed in the United Nations Single Convention on Narcotic Drugs (New York, 1961; amended 1972), the Convention on Psychotropic Substances (Vienna, 1971), and the Convention against Illicit Traffic in Narcotic Drugs and Psychotropic Substances (Vienna, 1988). These listings, which include more than 250 compounds and precursors, were created to facilitate their control and to limit their use. These compounds include, but are not limited to, cannabis, cocaine, heroin, amphetamines, LSD, ketamine, etc. [1,2,3,4]. Over one million seizures of illicit drugs are reported annually in Europe, where cannabis is the most commonly seized drug, accounting for over 70% of cases. Cocaine ranks second overall (9%), followed by amphetamines (5%), heroin (5%) and ecstasy (2%). It is estimated that about 7500 overdose deaths, involving at least one illicit or controlled drug, occurred in 2015 in the European Union (EU) [2].

For these reasons, the analysis of controlled drugs in forensic matrices, such as urine, blood, plasma, saliva, and even hair, is of paramount importance [5,6,7]; this includes the initial diagnosis of drug addiction, mandatory screening in some treatment programs and in the workplace, doping control, screening as a method of tracking drug effects over time, identification of the substance in case of an overdose, and determination of treatment compliance.

Drug analysis in forensic fluids usually involves four main steps, i.e., enrichment of the target substances from the biological matrices, separation from potential interferences, detection, and data handling. Nevertheless, analyses of biological samples are always challenging due to the very high complexity of these matrices. The presence of endogenous interferences, such as proteins in plasma, serum, and breast milk, as well as inorganic salts in urine, demands for sample treatment prior to instrumental analysis. Additionally, the target compounds are usually present in very low concentrations and, for this reason, sample preparation becomes crucial [8]. As a general rule, most sample preparation stages include an extraction step aiming to transfer the target analytes to a phase more compatible with the instrumental systems, concentrate the solutes if we are dealing with trace analysis, and elimination of undesirable interferences. Modern approaches to sample enrichment run towards the great simplification, miniaturization, easy manipulation of the analytical devices, high-throughput performance, automation, online coupling with instrumental systems, low sample-volume requirements, and the strong reduction or absence of toxic organic solvents in agreement with the green analytical chemistry principles [9,10,11,12].

In this regard, liquid- or sorbent-phase microextraction techniques have become prominent both in passive and active modes. The former includes methodologies such as liquid-phase microextraction (LPME), single-drop microextraction (SDME), hollow-fiber microextraction (HF-LPME), solvent bar microextraction (SBME), electromembrane microextraction (EME), parallel artificial liquid membrane extraction (PALME), and dispersive liquid–liquid microextraction (DLLME). On the other hand, the latter includes analytical technologies such as solid-phase microextraction (SPME), stir-bar sorptive extraction (SBSE), bar adsorptive microextraction (BAμE), thin film microextraction (TFME), solid-phase extraction (SPE) and micro-solid-phase extraction (µSPE), magnetic-solid-phase extraction (mSPE), molecularly imprinted solid-phase extraction (MISPE), immunoaffinity solid-phase extraction (IASPE), microextraction by packed sorbent (MEPS), and disposable pipette extraction (DPX). For more details on sample preparation for drug analysis, it is recommended to consult several reference books [13,14,15,16,17] and research review articles [5,6,7,8,9,10,11,12,18,19,20,21,22,23,24,25,26,27,28,29,30]. These miniaturized analytical approaches have been proposed for more than two decades to monitor controlled drugs in biological matrices, such as opioids and related compounds, cocaine and metabolites, amphetamine-type substances (ATS), cannabinoids, hallucinogens, dissociative drugs, and new psychoactive substances (NPS). However, limited manuscripts cover the broad range of applications of miniaturized techniques for sample preparation in the forensic context. Most review papers are dedicated to specific technique(s), the sorbent/liquid phase, a particular class of controlled drugs, or are in a target sample type [8,10,11,18].The present contribution focuses on an overview regarding the application of all microextraction-based techniques for screening-controlled drugs in the forensic context, including the important analytical characteristics and parameters of several applications. It also discusses the advantages and limitations of miniaturized sample preparation techniques in this context. We believe that the present contribution can facilitate researchers in choosing a particular analytical approach for their application goal.

## 2. Screening-Controlled Drugs by Microextraction-Based Techniques

### 2.1. Opioids and Related Substances

The classes of drugs known as opiates or opioids include a wide and diverse range of natural and synthetic chemical compounds derived from opium. These drugs have been used for centuries for the purpose of reducing pain; however, in some cases their effects may lead to overdose [31,32,33,34,35,36,37,38]. In England and Wales alone, heroin or morphine was connected to 1200 deaths registered in 2015 [2]. When administrated, most opioids undergo extensive varying degrees of phase 1 and 2 metabolism. Phase 1 metabolism usually precedes phase 2 metabolism, but this is not always the case. Phase 1 metabolism typically subjects the drug to oxidation or hydrolysis, whereas phase 2 metabolism conjugates the drug to hydrophilic substances, such as glucuronic acid, sulfate, glycine, or glutathione. The process of metabolism ends when the molecules are sufficiently hydrophilic to be excreted from the body. For more details regarding opioid metabolism it is advisable to consult the work published by Smith [39]. This information is relevant, especially considering that some precautions may be needed when trying to analyze these compounds. Depending on the target compounds and matrices, the hydrolysis of the glucurinated compounds, as well as protein precipitation, may be needed to increase the signal of the target compounds. For this reason, the determination of these compounds in biological matrices requires the development of reliable analytical methods in clinical, forensics, and research contexts.

Several applications have been developed for the analysis of opioids and related substances in forensic matrices using miniaturized sample preparation approaches. Most were based on headspace (HS) or direct immersion SPME and DLLME. Nevertheless, alternative techniques have been also proposed, including EME, HP-LPME, miniaturized SPE, MEPS, and SDME, among others. For example, Vlčková et al. [40] used a fast MEPS coupled directly to the mass spectrometry (MS) method for the determination of methadone in human urine. This approach achieved convenient detection limits (1.5 μg/L) and remarkable recoveries (~100%) using only 0.1 mL of sample. Another relevant work was published by Gonçalves et al. [41], using a very cost-effective technique (BAμE coated with activated carbons (ACs)) for the enrichment of morphine and codeine from human urine matrices. This sample preparation technique was combined with high-performance liquid chromatography–diode array detection (HPLC-DAD), which allowed the researchers to attain suitable detection limits (0.06–0.90 μg/L) and inter-day repeatability (≤8.0%). Ranjbari et al. [42] reported a work which combines DLLME with HPLC-UV/vis detection for the analysis of methadone in several human biological matrices, using only 0.5 mL of plasma and urine and 0.1 mL of saliva and sweat. This methodology presented very good inter-day repeatability (<6.4%) and accuracy levels (~100%). Habibi-Khorasani et al. [43] developed a molecularly imprinted polymer for SPME fibers for the selective enrichment of tramadol from brain tissues. The data achieved was very good, as well as the extraction yields (76.2–91.2%) and inter-day repeatability (≤8.2%), using just 2 g of sample. Table 1 summarizes with more detail the miniaturized applications discussed herein for the enrichment of opioids and related substances in forensic matrices, with emphasis on the key characteristics on the developed methodologies. Table S1 (Supplementary Materials) contains information regarding other techniques for the determination of these classes of compounds in the forensic context [44,45,46,47,48,49,50,51,52,53,54,55,56,57,58,59,60,61,62,63,64,65,66,67,68,69,70,71,72,73,74,75,76,77,78,79,80,81,82,83,84,85,86,87,88,89,90,91,92,93,94,95,96,97,98,99,100,101,102,103,104,105].

### 2.2. Stimulants and Related Substances

One of the most common controlled stimulant drugs is cocaine. It is a naturally occurring substance found in leaves of Erythroxylon coca (containing between 0.6% and 1.8% alkaloidal cocaine), a plant endogenous in South America, Mexico, Indonesia, and the West Indies [31,32,35,106]. By the turn of the 20th century, cocaine’s addictive properties became well-known, and nowadays, it is classified as a Schedule II drug in the United States of America (USA), owing to its high potential for abuse [107,108]. In 2016, an estimated 18.2 million people were described as cocaine users, of which there were more than 10,000 related deaths in the USA [109] and 100 in Turkey [110].

Several applications have been developed for the analysis of cocaine and related substances in forensic matrices using miniaturized sample preparation approaches. Most were based on µSPE, HS, or direct immersion SPME and HP-LPME approaches. Nevertheless, other techniques were also proposed, including EME, DLLME, and DPX. A fairly recent work developed by Rosado et al. for the rapid analysis (15 min) of cocaine and metabolites in urine matrices using MEPS and microwave derivatization followed by gas chromatography coupled to mass spectrometry (GC-MS) [111]. The methodology was fully validated and allowed for the quantification of the target compounds in real matrices. Another work employed HP-LPME for the enrichment of cocaine and metabolites from breast milk followed by derivatization and GC-MS analysis [112]. This methodology employed only 30 min of equilibrium time, allowing researchers to extract up to 67% of the target compounds. Sánches-Gonzáles et al. [113] developed a magnetic imprinted polymer using µSPE to selectively and quickly (~4 min) retain cocaine and its main metabolites from plasma samples. The optimized methodology was combined with liquid chromatography tandem mass spectrometry (LC-MS/MS) analysis, resulting in very low detection limits (0.013–0.36 pg/mL). Table 2 resumes with detail these miniaturized applications for the enrichment of cocaine and related substances in forensic matrices, with emphasis on the key characteristics on the developed methodologies. Table S2 (Supplementary Material) contains information regarding other techniques for the determination of these classes of compounds in the forensic context [52,84,99,100,104,114,115,116,117,118,119,120,121,122,123,124,125,126,127,128,129,130,131,132,133].

Another important class of stimulant drugs are amphetamine (AMP) and amphetamine-type substances (ATS), which include methamphetamine (MAMP), 3,4-methylenedioxymethamphetamine (MDMA, ecstasy), N-ethyl-3,4-methylenedioxyamphetamine (MDEA), and methylenedioxyamphetamine (MDA). These compounds are derived from ephedra (Ephedra sinica), a native plant from China and Mongolia [134,135]. Stimulants such as cocaine, amphetamines, MDMA, and cathinones are implicated in several overdose deaths in Europe. For instance, stimulant-related deaths in Turkey totaled 206 cases related to amphetamines and 166 cases related to MDMA in 2015 [2].

Several applications have been developed for the analysis of amphetamine and related substances in forensic matrices using miniaturized sample preparation approaches. Most were based on HS or direct immersion SPME, DLLME, and HP-LPME. Nonetheless, other techniques were also proposed, including EME, SDME, DPX, and dSPE, among others. Recently, Song and Yang [136] employed an electric field to accelerate the mass transfer of AMP and MAMP from urine to a single-drop extraction phase, resulting in recovery yields of up to 96%. Taghvimi et al. [137] introduced a metal organic framework-based carbon porous as an efficient dSPE for the enrichment of MAMP from urine samples. Maddadi et al. [138] developed a floating HP-LPME-based methodology for the extraction, preconcentration, and determination of methylphenidate in urine matrices. The results showed good inter-day repeatability levels (≤3.9%), using only 25 min of microextraction time. Abbasian et al. [139] developed a new SPME fiber based on multiwalled carbon nanotubes and ionic liquids for the separation and determination of methamphetamine and MDMA in human urine using a sol-gel preparation. The results showed high sensitivity (0.097–0.390 ng/mL) and suitable inter-day repeatability (≤7.0%). Table 3 summarizes in detail these miniaturized applications for the enrichment of amphetamine and related substances in forensic matrices, with emphasis on the key characteristics on the developed methodologies. Table S3 (Supplementary Material) contains information regarding other techniques for the determination of these classes of compounds in the forensic context [40,89,90,91,92,93,94,95,96,97,98,100,104,105,129,130,131,140,141,142,143,144,145,146,147,148,149,150,151,152,153,154,155,156,157,158,159,160,161,162,163,164,165,166,167,168,169,170,171,172,173,174,175,176,177,178,179,180,181,182,183,184,185,186,187,188,189,190,191,192,193,194,195,196,197,198,199,200].

### 2.3. Cannabinoids and Related Substances

Cannabis sativa, more commonly known as “marijuana”, is a hemp plant that grows freely throughout the world [31]. It is estimated that the global number of users of cannabis is around 182.5 million. The total number of cannabis seizures (including herd and resin) in 2014 was more than 7000 tons [1]. In the EU alone, herbal and resin cannabis accounted for around 69% of total seizures (568 tons) in 2016 [48].

The first chemical analysis of cannabis apparently was performed in 1821. Since then, studies have shown cannabis to be a complex plant, in which more than 400 individual chemical compounds have been identified. The most potent cannabinoid is Δ-9-tetrahydrocannabinol (THC) and other relevant ones include, Δ-8-tetrahydro-cannabinol (8-THC), cannabidiol (CBD), and cannabinol (CBN).

Several applications have been developed for the analysis of cannabinoids and related substances in biological matrices using miniaturized sample preparation approaches. Most were based on HS or direct immersion SPME and DPX. Nevertheless, other techniques were also employed, including MEPS, HF-LPME, and µSPE, among others. Anderson et al. [201] developed DPX containing a weak anionic exchange polymer to selectively extract 11 cannabinoids and metabolites from human urine samples. This methodology was followed by LC-MS/MS, resulting in suitable extraction efficiencies (up to 81%) and convenient detection limits (0.5–5.0 ng/mL). Sánchez-González et al. [202] developed a molecularly imprinted polymer (MIP) for μSPE to selectively retain THC, THC-COOH, and THC-OH from plasma and urine, followed by LC-MS/MS. To produce the MIP, the authors used THC-COOH as a template molecule, whereas ethylene glycol dimethacrylate was used as a functional monomer, divinylbenzene as a cross-linker, and 2,2′-azobisisobutyronitrile as an initiator. The optimized methodology allowed the researchers to attain high recovery yields (up to 94%) using only 12 min of equilibrium time. Using a conventional HS-SPME-based methodology, Silveira et al. [203] were able to selectively extract three cannabinoids from human breast milk. The microextraction step was followed by thermal desorption (TD) and GC-MS analysis, resulting in suitable sensitivity (10 ng/mL) using only 0.05 mL of sample. On the other hand, Emídio et al. [204] applied a HF-LPME-based methodology for the enrichment of the same cannabinoids from hair samples using GC-MS as instrumental system. The results indicated good detection limits (0.5–15 pg/mg) using only 10 mg of sample. Table 4 summarizes in detail these miniaturized applications for the enrichment of cannabinoids and related substances in forensic matrices, with emphasis on the key characteristics on the developed methodologies. Table S4 (Supplementary Material) contains information regarding other techniques for the determination of these classes of compounds in the forensic context [89,100,205,206,207,208,209,210,211,212,213,214,215,216,217,218,219].

### 2.4. Hallucinogens, Dissociative Drugs, and Related Substances

Hallucinogens are substances that promote hallucinations, a unique psychoactive effect, which are profound distortions in a person’s perceptions of reality. Hallucinogens can be found in some plants and mushrooms (or their extracts) or can be man-made, and they are commonly divided into two broad categories: classic hallucinogens, including lysergic acid diethylamide (LSD), 4-phosphoryloxy-N,N-dimethyltryptamine (psilocybin), peyote (mescaline), and dimethyltryptamine or ayahuasca (DMT); and dissociative drugs, including ketamine (KET), dextromethorphan, phencyclidine (PCP), and Salvia divinorum. When under the influence of either type of drugs, people often report experiencing rapid, intense emotional swings and seeing images, hearing sounds, and feeling sensations that are unreal [31,32].

To date, there has not been a microextraction-based approach dedicated solely to the determination of classic hallucinogens from forensic matrices, and very few have included these compounds as target analytes in their method development. Nevertheless, recently, Vicenti et al. [100] developed a very comprehensive methodology using DLLME combined with pressurized liquid extraction, and liquid chromatography coupled to high-resolution tandem mass spectrometry (LC-HRMS/MS) for the enrichment of over 60 drugs of abuse in hair, including mescaline. The results showed that the developed method allowed the researchers to attain recovery yields of up to 40% for mescaline using only 10 mg of sample. Table 5 resumes in detail most of the miniaturized applications for the enrichment of classic hallucinogens and related classes of compounds in the forensic context, with emphasis on the key characteristics of the developed methodologies.

As was mentioned before, another important class to consider is dissociative drugs, which include dextromethorphan, Salvia divinorum, PCP, and KET, in which the latter have been more largely abused than the other dissociative drugs.

PCP was synthesized in 1956 and was tested as an anesthetic because it had pronounced tranquilizing effects. With animals, it produced a general anesthesia that left them conscious but not feeling pain, even during surgery. In clinical trials of PCP with humans, however, some patients experienced hyperexcitability, delirium, and visual disturbances. For this reason, it was largely abandoned for human use [31].

KET was developed in 1962 during a search for a less problematic replacement for PCP. Due to its quick onset and short duration of action with only slight cardiorespiratory depression in comparison with other general anesthetics and the possibility of inhalation to maintain the anesthetic state, KET is a preferred drug for short-term surgical procedures in veterinary and human medicine, especially in children [221]. Several applications have been developed for the analysis of cannabinoids and related substances in forensic matrices using miniaturized sample preparation approaches. Most were based on HS or direct immersion SPME, DLLME, and HF-LPME. Nevertheless, other techniques were also employed, including MEPS, SDME, µSPE, and high-throughput bar adsorptive microextraction (HT-BAµE), among others. One of the first reports using microextraction-based approaches to determine dissociative drugs in forensic matrices was developed by Ishii et al. [222]. These authors used HS-SPME to selectively extract PCP from urine and blood samples followed by thermal desorption and gas chromatography with surface ionization detection (TD/GC-SID) analysis. The results showed that recoveries were up to 48% with limits of detection between 0.25 and 1.0 ng/mL, depending on the matrix type. Casari and Andrews [195] developed a floating HP-LPME-based methodology for the extraction, preconcentration, and determination of PCP and other compounds from urine samples. This approach employed only 2 µL of chloroform as extraction solvent, resulting in suitable sensitivity with convenient limits of detection (70 ng/mL). On the other hand, Meng et al. [92] decided to compare HF-LPME and ultrasound-assisted DLLME methodologies combined with GC-MS for the determination of selected drugs of abuse, including KET, in biological samples. The results show similar detection limits, extraction efficiencies and inter-day repeatability levels, although the former employed a smaller amount of organic solvent. More recently, a HT-BAµE methodology was developed to monitor KET and its main metabolite (norketamine) in urine matrices, followed by GC-MS. The developed apparatus allows for the extraction and desorption of the target compounds in up to 100 urine samples simultaneously, resulting in an assay time of only 0.45 min/sample [223]. Table 6 summarizes in detail these miniaturized applications for the enrichment of dissociative drugs and related substances in forensic matrices, with emphasis on the key characteristics on the developed methodologies. Table S5 (Supplementary Material) contains information regarding other techniques for the determination of these classes of compounds in the forensic context [89,90,98,100,105,120,129,130,145,157,164,174,194,196,224,225,226,227,228,229,230,231,232,233,234].

## 3. Future Trends in the Forensic Context

### 3.1. New Psychoactive Substances (NPS)

**Figure 1:**
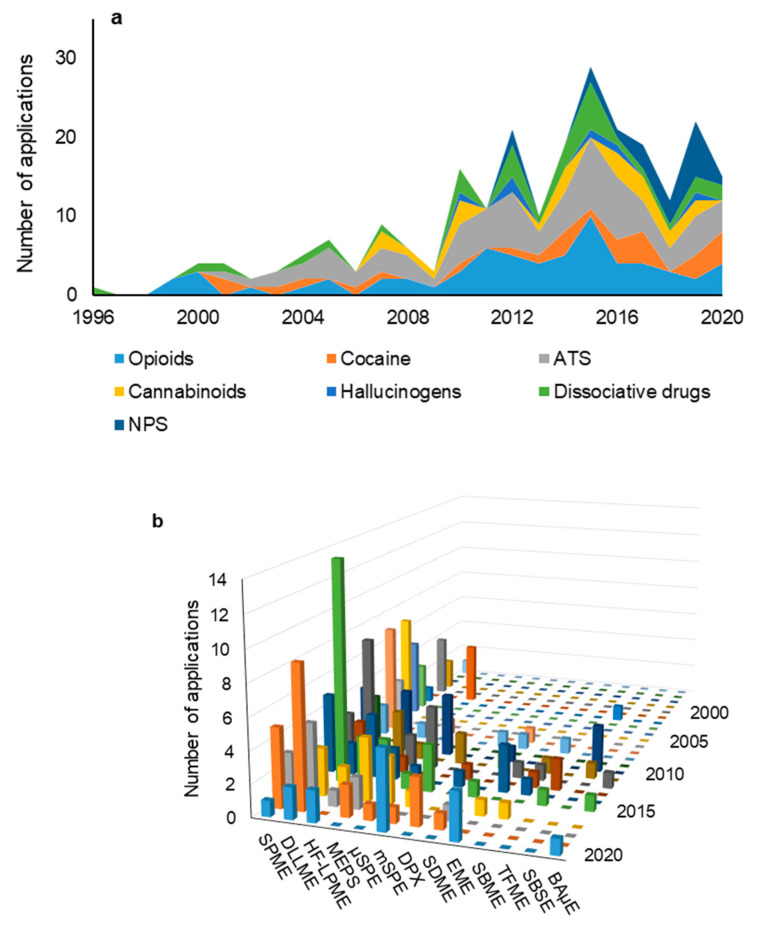
Number of applications for screening-controlled drugs in forensic matrices over the last quarter of century sorted by the type of priority substances (**a**) and microextraction-based techniques (**b**). The data reported for 2020 only includes published articles until the end of June.

Nowadays, the illicit drug market has become more versatile than ever, where not only the classic drugs (i.e., cocaine, cannabis, heroin, etc.) are sold, but also NPS. Although some of these compounds are not really “new” since they were developed many decades ago, most (<85%) appeared only in the last decade, being sold in smart shops and on the Internet as innocuous products designed “not for human consumption” [235]. A NPS is defined as “a new narcotic or psychotropic drug, in pure form or in preparation, that is not controlled by the United Nations drug conventions, but which may pose a public health threat” comparable to that substances listed in these conventions [236]. Many of them are traded as “legal” replacements to established controlled drugs such as cannabis, heroin, benzodiazepines, cocaine, amphetamines, and MDMA. In 2018, the number of NPS controlled by the EMCDDA reached a total of over 730 substances that have been detected in a wide range of different products, including synthetic cannabinoids, opioids, benzodiazepines, arylcyclohexylamines, synthetic cathinones, and phenethylamines. However, understanding the epidemiology of NPS remains poor. This includes problems with estimating the prevalence of use of new substances, which can be a complex and hard task because of the large number of substances and products that are available, but also because of the highly dynamic nature of the market. In many cases, individuals do not actually know what new substance they are using, while in other cases they may not even realize that they are using a new one. Therefore, the detection of NPS, and especially their metabolites in the forensic context, will remain a very hot topic for years to come. Figure 1a shows the number of applications for screening-controlled drugs in forensic matrices over the last quarter of a century, including opioids, cocaine, ATS, cannabinoids, hallucinogens, dissociative drugs, and NPS, and the figure highlights that monitoring the latter class of substances has grown in the last decade. For these reasons, several applications have been proposed for the analysis of NPS and related substances in the forensic context using innovative sample preparation approaches. Odoardi et al. [237] developed a comprehensive screening method for 78 substances in whole blood using DLLME combined with ultra-high-performance liquid chromatography coupled to tandem mass spectrometry (UHPLC-MS/MS). The results showed excellent recovery yields (~100%) with suitable inter-day repeatability levels (<15%). The target analytes included cathinones, synthetic cannabinoids, phenethylamines, piperazines, KET, analogues, benzofurans, tryptamines, and some of their metabolites. The data showed that high recovery rates (up to 110%) and low detection limits (0.2 ng/mL) were achieved. More recently, Bianchi et al. [238] combined MEPS and desorption electrospray ionization high-resolution mass spectrometry (DESI-HRMS) for the analysis of selected synthetic cathinones and cannabinoids in oral matrices. The results showed that good quantification limits were achieved (0.25–0.5 mg/L) using only 25 µL of sample. Additionally, a very simple and effective BAµE-based approach was also proposed for the enrichment of mitragynine from urine samples, followed by liquid desorption and HPLC-DAD analysis [239]. Table 7 summarizes in detail the main miniaturized microextraction applications for the enrichment of NPS and related substances in forensic matrices, with emphasis on the key characteristics on the developed methodologies. Table S6 (Supplementary Material) contains additional information regarding other techniques for the determination of NPS in the forensic context [99,100,128,131,143,197,219,238,239,240,241,242,243,244,245,246].

### 3.2. Comparison of the Top-Eight Most Applied Microextraction-Based Techniques

Miniaturized analytical techniques present many advantages over conventional sample preparation approaches. For this reason, they have been proposed in the last two decades for screening-controlled drugs in the forensic context, such as opioids and related compounds, cocaine, metabolites, ATS, cannabinoids, hallucinogens, dissociative drugs, and NPS. Figure 1b shows the total number of applications dedicated for screening-controlled drugs in forensic matrices over the last two decades, sorted by the microextraction-based techniques. Most techniques are based on SPME, DLLME, HF-LPME, and MEPS, but also µSPE and BAµE (among others); good examples can be consulted in the bibliography. From all these techniques, SPME and DLLME represent more than 50% of the miniaturized microextraction-based applications in the forensic context, and they were mostly applied to monitor opioids and ATS in biological matrices. Nonetheless, the other approaches have also gained some notoriety over the last few years, once they presented additional interesting characteristics. However, if we evaluate and compare the adequacy of the main characteristics of the top-eight most frequently applied microextraction techniques for screening-controlled drugs in the forensic context, several advantages and limitations can be noted, as summarized in Table 8.

In any analytical framework, these relevant characteristics condition the sample preparation stage (whether it is user-friendly, environmentally acceptable, reusable, cost-effective, and allows for routine work), the instrumental systems (whether the online coupling is possible and comprehensive), as well as the analytical performance (namely, if the enrichment factor, recovery yields, and level of precision are favored). In addition, the assessment of these characteristics is very important in particular for the beginners who intend to select and start to apply microextraction-based techniques for screening-controlled drugs in any forensic context. For instance, the sorbent-based microextraction techniques have been much more applied for screening-controlled drugs in the forensic context than the liquid-based ones, in which methodologies such as DLLME, HP-LPME, and SDME standing out mainly for their very low cost. Nevertheless, the sorbent-based techniques, such as SPME, SBSE, BAµE, MEPS, and µSPE, have been more widely employed since they present much more advantageous characteristics in any analytical framework. Even so, the passive or non-exhaustive sampling techniques (SPME, SBSE, and BAµE) have been proposed more than the active or exhaustive sampling ones (MEPS and µSPE), because they present interesting features.

Therefore, we can carry out a detailed analysis, as shown in Table 8, on the most relevant or “extremely suitable” (+++) characteristics, presented by the main passive sorbent-based methodologies. In general, SPME is the most well-established microextraction technique, largely used in the forensic context, because it presents several advantages: it is an eco-friendly approach (i.e., a solventless or solvent-free process) and can be applied for routine work allowing for online coupling and automation, particularly with GC systems. On the other hand, SBSE and BAµE are user-friendly approaches that show great simplicity of handling. Furthermore, the former present wide reusability and high enrichment factors (such as SPME), whereas the latter is very cost-effective and comprehensive (like µSPE), because it can be applied to a large number of classes of compounds having widened polarity and allows for coupling with both GC and HPLC systems. In addition to other distinct characteristics that it presents, the BAµE devices can be lab-made, which is a remarkable advantage over the remaining sorbent-phase microextraction techniques. Furthermore, microextraction techniques such as BAµE allow for the high-throughput approach (HT-BAµE), which is an asset for the routine analytical work, as discussed before.

## 4. Concluding Remarks

The need for analytical methodologies that currently meet the high selectivity, sensitivity, and the principles of green analytical chemistry that are required by forensic science, pushes for the development of novel microextraction-based techniques.

Although the application of these techniques for screening-controlled drugs in forensic matrices is not yet very extensive, several works have shown that they are credible alternatives as analytical methodologies. Even so, these miniaturized techniques still need further improvement in clinical and toxicological context, although potential alternatives have shown several advantages in comparison to the well-established reference methods, because many of them allow for fast response in routine work. This is particularly important for the rapid detection of the increasing occurrence of NPS worldwide, since the development and implementation of faster alert systems is critical for legal and judicial authorities.

## Figures and Tables

**Table 1:** Microextraction-based approaches for the analysis of opioids and related substances in the forensic matrix.

Drugs	Matrix	Sample Amount	Sample Pretreatment	Microextraction Technique	Optimized Experimental Conditions	Instrumental System	LOD (μg/L)	Absolute Recovery (%)	Precision (%)	Ref.
**MTD**	Urine	0.1 mL	-	MEPS	Sorbent: C8 Activation: 100 μL MeOH × 3 Conditioning: 100 μL H_2_O × 3 Samples aspirated and discarded × 5 Washing: 100 μL H_2_O × 2 + 100 μL of 5 % MeOH × 1 Elution: 50 μL of 0.1% COOH in MeOH	MS/MS	1.5	91.7–106.7	≤11.1	[40]
**TMD**	Rabbit brain tissue	2 g	Solid–liquid extraction Centrifugation Evaporation Dilution	DI-SPME	Washing: 1 mL acetone:ACN (3:1, *v*/*v*) Eluting step: 0.2 mL HCl (1 mol/L):MeOH (1:1, *v*/*v*)	HPLC-UV	1	76.2–91.2	≤8.2	[43]
**COD** **MOR**	Urine	2 mL	Filtration Sonication Dilution	BAμE	Coating: ACs Extraction: 2.5 h, 1000 rpm (pH 7) LD: MeOH/ACN (1:1, 1.5 mL), 30 min Evaporation to dryness and redissolution	HPLC-DAD	0.06–0.90	38.4–41.3	≤8.0	[41]
**MTD**	Urine Plasma Saliva Sweat	Urine and plasma: 0.5 mL Saliva and sweat: 0.1 mL	Urine and plasma: Centrifugation Filtration Dilution Saliva and sweat: Dilution	DLLME	Sample at pH 10 DS: MeOH (2.5 mL) ES: CHCl_3_ (200 μL) Centrifugation Sediment dried and redissolved	HPLC-UV	4.9 7.3 25.12 24.85	98.6–100.3	≤6.4	[42]

ACN: acetonitrile; AP: acceptor phase; BAμE: bar adsorptive microextraction; C8: octyl silica; CHCl_3_: chloroform; COD: codeine; COOH: formic acid; DI-SPME: direct immersion solid-phase microextraction; DS: disperser solvent; ES: extraction solvent; H_2_O: ultra-pure, distilled, or double-distilled water; HPLC: high-performance liquid chromatography; LD: liquid desorption; LDC: lidocaine; LOQ: limit of quantification; MeOH: methanol; MEPS: microextraction by packed sorbent; MS/MS: tandem mass spectrometry; MTD: methadone; NaCl: sodium chloride; TD: thermal desorption; TMD: tramadol; UV: ultraviolet.

**Table 2:** Microextraction-based approaches for the analysis of cocaine and related substances in the forensic matrix.

Drugs	Matrix	Sample Amount	Sample Pretreatment	Microextraction Technique	Optimized experimental Conditions	Instrumental System	LOD (μg/L)	Absolute Recovery (%)	Precision (%)	Ref.
**CCE** **COC** **NC**	Breast milk	0.5 mL	pH adjustment Salt addition	HF-LPME	Extraction: 30 min (2400 rpm, pH 9.0, 25% NaCl) SLM: 1-octanol AP: 0.4 mol/L HCl Evaporation Derivatization	GC-MS	5–7	32.0–67.4	≤15.9	[112]
**BE** **COC** **ECGME**	Urine	0.2 mL	Centrifugation Dilution pH adjustment	MEPS	Sorbent: 80% C8 + 20% SCX Conditioning: 250 μL MeOH × 1 + 250 μL 0.1% COOH × 1 Samples aspirated and discarded × 6 Washing: 50 μL 0.1% COOH × 4 + drying Elution: 100 μL of 1% NH_4_OH in MeOH × 4 Evaporation to dryness and derivatization	GC-MS	25 (LLOQ)	14.5–83.3	≤14.38	[111]
**BE** **CCE** **COC** **ECGME**	Plasma	0.1–1.0 mL	pH adjustment Dilution	μSPE	Sorbent: MMIP Extraction: 4 min (20 °C, 100 rpm, 0.2 mL n-C6) Elution: 2 mL DCM/IPA/NH_4_OH (75:20:5, *v*:*v*:*v*), ultrasound irradiation (5 min) Evaporation to dryness Redissolved with 0.04 mL of 2 mM C_2_H_7_NO_2_ in MeOH	LC-MS/MS	0.000013–0.00036	91–102	≤10	[113]

µSPE: micro-solid-phase extraction; ACN: acetonitrile; AP: acceptor phase; BE: benzoylecgonine; BN: benzoylnorecgonine; CCE: cocaethylene; CHCl3: chloroform; CME: capillary microextraction; COC: cocaine; COOH: formic acid; ECG: ecgonine; ECGME: ecgonine methyl ester; GC: gas chromatography; HF-LPME: hollow-fiber liquid-phase microextraction; IPA: isopropanol/2-propanol; LC: liquid chromatography; MeOH: methanol; MEPS: microextraction by packed sorbents; MMIP: magnetic molecularly imprinted polymer; MS/MS: tandem mass spectrometry; MS: mass spectrometry; NaCl: sodium chloride; NC: norcocaine; n-C6: n-hexane; NCE: norcocaethylene; PDMS: polydimethylsiloxane; SCX: silica-based cationic exchange polymer; SLM: supported liquid membrane.

**Table 3:** Microextraction-based approaches for the analysis of amphetamine and related substances in the forensic matrix.

Drugs	Matrix	Sample Amount	Sample Pretreatment	Microextraction Technique	Optimized Experimental Conditions	Instrumental System	LOD (μg/L)	Absolute Recovery (%)	Precision (%)	Ref.
**AMP** **4-MAMP**	Urine	1 mL	Filtration Dilution pH adjustment Derivatization	EE-SDME	Solvent: DCM (2 μL) Extraction: 4 min (−4 V, pH 7)	GC-FID	0.14–0.27	82.7–96.2	≤12.8	[136]
				HS-SPME	Coating: PDMS-DVB Extraction: pH 7, 40 min (600 rpm, 60 °C) TD: 5 min, 250 °C		0.05–0.09	90.7-112.5	≤8.5	
**MET**	Urine	5 mL	pH adjustment Centrifugation	dSPE	Sorbent: ZIFs (40 mg) Extraction: 5 min (2000 rpm) Centrifugation Desorption: 400 μL MeOH (sonication for 10 min) Centrifugation	HPLC-UV	10	99.83	≤5.1	[137]
**MPH**	Urine	2.5 mL	Dilution Centrifugation pH adjustment NaCl addition	SBME	Extraction: 25 min (650 rpm, pH 11.6, 25 °C, 30% NaCl (*w*/*v*)) SLM: 1-octanol AP: pH 4.0, 30 μL	HPLC-UV	15	n.a.	≤3.9	[138]
**MDMA** **MET**	Urine	2 mL	NaCl addition pH adjustment	HS-SPME	Coating: MWCNTs/ILs Extraction: pH 11, 20 min, 20% NaCl (*w*/*v*) (500 rpm, 80 °C) TD: 4 min, 250 °C	GC-FID	0.097–0.39	n.a.	≤7.0	[139]

4-MAMP: 4-methylamphetamine; AMP: amphetamine; AP: acceptor phase; DCM: dichloromethane; dSPE: dispersive solid-phase extraction; DVB: divinylbenzene; EE-SDME: enhanced single-drop microextraction; FID: flame ionization detector; GC: gas chromatography; HPLC: high-performance liquid chromatography; HS-SPME: headspace solid-phase microextraction; IL: ion liquid; MDMA: 3,4-methylenedioxymethamphetamine; MeOH: methanol; MET: methamphetamine; MPH: methylphenidate; MWCNT: multiwalled carbon nanotube; NaCl: sodium chloride; PDMS: polydimethylsiloxane; SBME: solvent bar microextraction; SDME: single-drop microextraction; SLM: supported liquid membrane; TD: thermal desorption; UV: ultraviolet; ZIFs: zeolitic imidazolate frameworks.

**Table 4:** Microextraction-based approaches for the analysis of cannabinoids and related substances in the forensic matrix.

Drugs	Matrix	Sample Amount	Sample Pretreatment	Microextraction Technique	Optimized Experimental Conditions	Instrumental System	LOD (µg/L)	Absolute Recovery (%)	Precision (%)	Ref.
**THC** **CBN** **CBD**	Breast milk	0.05 mL	Dilution pH adjustment	HS-SPME	Sorbent: PDMS Extraction: 70 °C, 40 min, 25% NaCl TD: 250 °C	GC-MS	10.0	n.a.	≤13.3	[203]
**THC** **11-OH-THC** **THC-COOH** **CBN** **CBD** **THCAA** **CBG** **THCV** **THC-gluc** **THC-COOH-gluc**	Urine	0.2 mL	Centrifugation Dilution Protein precipitation pH adjustment	DPX	Sorbent: WAX Samples aspirated × 4 Upper layer diluted and centrifuged	LC-MS/MS	0.5–5.0	42.4–81.5	≤14.3	[201]
**THC** **CBN** **CBD**	Hair	10 mg	Washing Alkaline digestion Evaporation to dryness Dilution	HF-LPME	Extraction: 20 min (pH 14, 6.8% NaCl, 600 rpm) SLM: butyl acetate AP: 20 μL butyl acetate	GC-MS	0.5–15 pg/mg	4.4–8.9	≤13.7	[204]
**THC** **11-OH-THC** **THC-COOH**	Plasma and urine	Urine: 1 mL Plasma: 0.1 mL	Dilution pH adjustment	μSPE	Sorbent: MIP Conditioning: 5 mL of 0.1 M/0.1 M phosphate/NaOH buffer solution (pH 6.0) for 10 min Extraction: 150 rpm (12 min, 40 °C) Rinsing: 5 mL of 0.1 M/0.1 M phosphate/NaOH buffer solution at pH 6.0 for rinsing (ultrasound assistance, 37 kHz, 325 W, 8 min) Elution: 2 mL of MeOH/aqueous acetic acid (90:10, *v*/*v*) through sonication (37 kHz, 325 W, 6 min)	HPLC-MS/MS	Urine: 0.14–0.16 Plasma: 0.11–0.15	87–94	Urine: ≤6 Plasma: ≤11	[202]

µSPE: micro-solid-phase extraction; 11-OH-THC: 11-hydroxy-9-tetrahydrocannabinol; ACN: acetonitrile; AP: acceptor phase; CBD: cannabidiol; CBN: cannabinol; DPX: disposable pipette extraction; GC: gas chromatography; HP-LPME: hollow-fiber liquid-phase microextraction; HPLC: high-performance liquid chromatography; HS-SPME: headspace solid-phase microextraction; MeOH: methanol; MIP: molecular imprinted polymer; MS/MS: tandem mass spectrometry; MS: mass spectrometry; NaCl: sodium chloride; PDMS: polydimethylsiloxane; SLM: supported liquid membrane; THC: 9-tetrahydrocannabinol; THCAA: 9-tetrahydrocannabinolic acid; THC-COOH: 11-nor-9-carboxy-9-tetrahydrocannabinol; THC-COOH-gluc: 11-nor-9-carboxy-9-tetrahydrocannabinol-glucuronide; THC-gluc: 9-tetrahydrocannabinol-glucuronide; THCV: 9-tetrahydrocannabiverin; THCV-COOH: 11-nor-9-carboxy-9-tetrahydrocannabiverin; WAX: weak anionic exchange.

**Table 5:** Microextraction-based approaches for the analysis of hallucinogenic drugs and related substances in the forensic matrix.

Drugs	Matrix	Sample Amount	Sample Pretreatment	Microextraction Technique	Optimized Experimental Conditions	Instrumental System	LOD (µg/L)	Absolute Recovery (%)	Precision (%)	Ref.
**LSD**	Blood	0.5 mL	Protein precipitation Centrifugation Dilution Salt addition pH adjustment	DLLME	DS: 250 µL MeOH ES: 100 μL chloroform Rapid injection Sonication (1 min) Centrifugation (4000 rpm, 5 min) Infranatant collected, evaporated, and redissolved	UPLC-MS/MS	0.5	90–127	≤15	[90]
**LSD**	Urine	4 mL	Dilution Salt addition pH adjustment	DLLME	DS: 1505 μL ACN ES: 606 μL CH_2_Br_2_ Extraction: 30% NH_3_, pH ≥ 11.5 Rapid injection Centrifugation (9500 rpm,5 min) Infranatant collected, evaporated, and redissolved	CE-UV	3.9–6.3	80.3	≤12.0	[164]
**Psylocibin** **Mescaline**	Oral fluid	0.090 mL	Centrifugation pH adjustment Dilution	μSPE	Sorbent: C18 Extraction: 15 min (30 °C, 200 rpm) Washing: 100 µL H_2_O Elution: 100 µL MeOH containing 10 mM of COOH	LC-MS/MS	0.07–0.1	61–64	≤9	[129]
**Mescaline** **Psylocibin**	Urine Plasma	Urine: 0.090 mL Plasma: 0.180 mL	Urine samples were diluted Sonication Centrifugation pH adjustment Dilution	μSPE	Sorbent: C18 Washing: 100 µL H_2_O Elution: 100 µL MeOH containing 10 mM of COOH	LC-MS/MS	0.3–1.4	57–66	≤7	[130]
**Muscimol** **Tryptamine** **Tryptophan**	Urine	2 mL	Dilution Salt addition pH adjustment	HF-LPME	SLM: DEHPA in DHE AP: HCl 0.1 mol/L Extraction: 60 min (800 rpm, pH 5, 0.001% NaCl)	HPLC-UV	0.7–17	n.a.	≤10.2	[220]
**Mescaline**	Hair	10 mg	Washing Digestion PLE extraction Evaporation Redissolution Centrifugation Dilution pH adjustment Salt addition	PLE-DLLME	DS: 500 µL 2-propanol ES: 200 µL chloroform Extraction: 24% NaCl, pH 11.0, 10% iso-propanol Sonication (10 min) Centrifugation (9000 rpm, 5 min, 3 °C) Infranatant collected, evaporated, and redissolved	LC-HRMS/MS	0.1 pg/mg	39	≤19	[100]

µSPE: micro-solid-phase extraction; ACN: acetonitrile; C18: octadecyl silica; CE: capillary electrophoresis; CH_2_Br_2_: dibromo methane; COOH: formic acid; DEHPA: di-(2-ethylhexyl)phosphoric acid; DHE: dihexylether; DLLME: dispersive liquid–liquid microextraction; HF-LPME: hollow-fiber liquid-phase microextraction; HPLC: high-performance liquid chromatography; HRMS/MS: high-resolution tandem mass spectrometry; LC: liquid chromatography; LSD: lysergic acid diethylamide; MeOH: methanol; MS/MS: tandem mass spectrometry; MS: mass spectrometry; NaCl: sodium chloride; NH_3_: ammonia; PLE: pressurized liquid extraction; UPLC: ultra-performance liquid chromatography; UV: ultraviolet.

**Table 6:** Microextraction-based approaches for the analysis of dissociative drugs and related substances in the forensic matrix.

Drugs	Matrix	Sample Amount	Sample Pretreatment	Microextraction Technique	Optimized Experimental Conditions	Instrumental System	LOD (µg/L)	Absolute Recovery (%)	Precision (%)	Ref.
**PCP**	Urine Whole blood	1 mL	Protein precipitation pH adjustment Salt addition	HS-SPME	Coating: PDMS Extraction: 30 min, 50% K_2_CO_3_ (*w*/*v*) (900 rpm, 90 °C) TD: 250 °C	GC-SID	0.25–1.0	9.3–47.8	≤27	[222]
**PCP**	Urine	2 mL	Filtration pH adjustment	SDME	Solvent: chloroform (2 μL) AP: (pH 10.5) Extraction: 8 min, 0.1 M NaOH	GC-PDHID	70	n.a.	≤16.2	[195]
**KET**	Urine Blood	1 mL	Dilution pH adjustment	HF-LPME	SLM: toluene AP: toluene (10 μL) Extraction: 10 min (500 rpm, pH 13.0, 30 °C)	GC-MS	2.5	81.3–98.6	≤4.5	[92]
				DLLME	ES and DS: 100 μL toluene Sonication (3 min) and manual shaking Centrifugation (10,000 rpm, 3 min) Supernatant collected For blood samples, 10 mg of NaCl was added to break emulsion		1.5–2.5	87.3–103.4	≤3.5	
**KET** **NorKET**	Urine	0.5 mL	Centrifugation Acid hydrolysis pH adjustment Dilution	HT-BAµE	Sorbent: NVP-DVB Extraction: 30 min (1800 rpm) pH 11.0 LD: sonication with 100 µL MeOH (15 min)	GC-MS	1.0	84.9–105.0	≤12.6	[223]

AP: acceptor phase; DLLME: dispersive liquid–liquid microextraction; DS: dispersion solvent; ES: extraction solvent; GC: gas chromatography; GC-SID: gas chromatography with surface ionization detection; HF-LPME: hollow-fiber liquid-phase microextraction; HS-SPME: headspace solid-phase microextraction; K_2_CO_3_: potassium carbonate; LC: liquid chromatography; MS: mass spectrometry; NaCl: sodium chloride; NaOH: sodium hydroxide; NVP-DVB: n-vinylpyrrolidone-divinylbenzene co-polymer; PCP: phencyclidine; PDHID: pulsed discharge helium ionization detector; PDMS: polydimethylsiloxane; SDME: single-drop microextraction; SLM: supported liquid membrane; TD: thermal desorption; UV: ultraviolet.

**Table 7:** Microextraction-based approaches for the analysis of new psychoactive substances (NPS) and related substances in the forensic matrix.

**Drugs**	**Matrix**	**Sample Amount**	**Sample Pretreatment**	**Microextraction Technique**	**Optimized Experimental** **Conditions**	**Instrumental System**	**LOD** **(μg/L)**	**Absolute Recovery (%)**	**Precision** **(%)**	**Ref.**
**Synthetic cathinones** **MPD** **Synthetic cannabinoid** **UR-144, JWH-250, JWH-200, JWH-122, JWH- 019, AM-2201, JWH-081, HU-211, CP47497**	Oral fluid	0.025 mL	Dilution Centrifugation pH adjustment	MEPS	Sorbent: C18 Conditioning: 100 μL MeOH + 100 μL H_2_O Samples aspirated × 5 (50 µL) Elution: 50 μL DCM/IPA/NH_4_OH × 25 Cleaning: 50 μL × 10	DESI-HRMS	0.25–0.5 mg/L (LLOQ)	n.a.	<19.4	[238]
**Mitragynine**	Urine	1 mL	Dilution	BAµE	Sorbent: NVP Extraction: 4 h (1300 rpm), pH 5.5 Elution: 200 μL MeOH/ACN (1:1, *v*:*v*) under sonication (10 min)	HPLC-DAD	0.1	103	≤15	[239]
**Synthetic cannabinoids** **AM-2201, AM-2233, AM-694, CB-13, JWH-007, JWH-019, JWH-015, JWH-018, JWH-030, JWH-073, JWH-081, JWH-098, JWH-122, JWH-147, JWH-200, JWH-201, JWH-250, JWH-251, JWH-307, JWH-398, RCS4, JWH-018 4OH indole, JWH- 018 5OH pentyl, JWH-018-COOH, JWH-073 4OH butyl, JWH-073 5OH indole, JWH-073 COOH, JWH-250 5OH pentyl** **Synthetic cathinones** **4-FAMP, 4-MEC, BL, BPD, CAT, EL, EPN, HML, HMO, MBDB, MDAI, MDPV, MPD, MD, ML, 4- MTA, NM-2-AI, PD, PL** **Piperaine derivatives** **BZP, mCPP**	Blood	0.5 mL	Protein precipitation Centrifugation Dilution Salt addition pH adjustment	DLLME	DS + ES: 350 μL of CHCl_3_/MeOH 1:2.5 (*v*:*v*), Rapid injection Sonication (2 min) Centrifugation (4000 rpm,5 min) Infranatant collected, evaporated, and redissolved	UHPLC-MS/MS	0.2	4–110	n.a.	[237]

µSPE: micro-solid-phase extraction; 25I-NBOMe: 4-iodo-2,5-dimethoxy-N-[(2-methoxyphenyl)methyl]-benzeneethanamine; 2-CB: 4-bromo-2,5-dimethoxyphenethylamine; 2C-E: 4-ethyl-2,5-dimetoxiphenethylamine; 2C-H: 2,5-dimethoxyphenethylamine; 2C-T-4: 2,5-dimethoxy-4-isopropylthiophenethylamine; 2C-T-7: 2-[2,5-dimethoxy-4-(propylsulfanyl)phenyl]ethan-1-amine; 2-FAMP: 2-fluoroamphetamine; 2-FMAMP: 2-fluoromethamphetamine; 2-FMC: 2-fluoromethcathinone; 2-MMC: 2-methoxymethcathinone; 3,4-DMMC: 3,4-dimethylmethcathinone; 3-FAMP: 3-fluoroamphetamine; 3-FEAMP: 3-fluoroethamphetamine; 3-MMAMP: 3-methoxymethamphetamine; 3-MMC: 3-methylmethcathinone; 4-CECAT: 4-chloroethcathinone; 4-CMCAT: 4-chloromethcathinone; 4-FAMP: 4-fluoroamphetamine; 4-FMAMP: 4-fluoromethamphetamine; 4-FMC: 4-fluoromethcathinone; 4-MEC: 4-methylethcathinone; 4-MeMABP: 4-methylbuphedrone; 4-MEPE: 4-methylephedrine; 4-MTA: 4-methylthioamphetamine; 6-APB: 6-(2-aminopropyl)benzofuran; AB-005: [1-[(1-methyl-2-piperidinyl)methyl]-1H-indol-3-yl](2,2,3,3-tetramethylcyclopropyl)-methanone; ACN: acetonitrile; AH-7921: 3,4-dichloro-N-{[1-(dimethylamino)cyclohexyl]methyl}benzamide; AM-1220: [1-[(1-methyl-2-piperidinyl)methyl]-1H-indol-3-yl]-1-naphthalenyl-methanone; AM-2201 4OH pentyl: AM-2201-N-(4-hydroxypentyl); AM-2201 metabolite: (1-(5-fluoro-4-hydroxypentyl)-1H-indol-3-yl)(naphthalen-1-yl)methanone; AM-2201: 1-[(5-fluoropentyl)-1H-indol-3-yl]-(naphthalen-1-yl)methanone; AM-2233: (2-iodophenyl)[1-[(1-methyl-2-piperidinyl)methyl]-1H-indol-3-yl]-methanone; AM-694: [1-(5-fluoropentyl)-1H-indol-3-yl](2-iodophenyl)-methanone; BL: butylone; BPD: buphedone; BPE: buphedrine; BPN: buprenorphine; bromo-dragonfly: 8-bromo-α-methyl-benzo [1,2-b:4,5-b’]difuran-4-ethanamine, monohydrochloride; BZP: 1-benzylpiperazine; C18: octadecyl silica; C8: octyl silica; CAT: cathinone; CB13: 1-naphthalenyl[4 -(pentylox)-1-naphthalenyl]-methanone; CHCl_3_: chloroform; COOH: formic acid; CP47497: [(1R,3S)-3-hydroxycyclohexyl]-5-(2-methyl-2-octanyl)phenol); CP47497-C8: C8 homologue of CP47497; DAD: diode array detection; DCA: dodecyl acetate; DCM: dichloromethane; DCP: dichloropane; DECAT: diethylcathinone; DESI: desorption electrospray ionization; DI-SPME: direct immersion solid-phase microextraction; DLLME: dispersive liquid–liquid microextraction; DMCAT: N,N-dimethylcathinone; DS: disperser solvent; DVB: divinylbenzene; ECAT: ethylcathinone; ECATEPE: ethylcathinone ephedrine; EDDP: 2-ethylidene-1,5-dimethyl-3,3-diphenylpyrrolidine; EL: ethylone; EPN: ethylphenidate; ES: extraction solvent; ETCAT: ethcathinone; FPD: flephedrone; GC: gas chromatography; H_2_O: distilled, deionized, or ultra-pure water; HCl: hydrochloric acid; HML: harmaline; HMO: harmalol; HPLC: high-performance liquid chromatography; HRMS: high resolution mass spectrometry; HU-211: dexanabinol; IMS: ion mobility spectroscopy; IPA: isopropanol/2-propanol; JWH-007: (2-methyl-1-pentyl-1H-indol-3-yl)-1-naphthalenyl-methanone; JWH-015: (2-methyl-1-propyl-1H-indol-3-yl)-1-naphthalenyl-methanone; JWH-018 4OH indole: JWH-018-4-hydroxyindole; JWH-018 4OH pentyl: JWH-018-4-hydroxypentyl; JWH-018 5OH pentyl: JWH-018-5-hydroxypentyl; JWH-018-COOH: JWH-018 N-pentanoic acid; JWH-019: (1-hexyl-1H-indol-3-yl)-1-naphthalenyl-methanone; JWH-030: 1-naphthalenyl(1-pentyl-1H-pyrrol-3-yl)-methanone; JWH-073 4OH butyl: JWH-073-4-hydroxybutyl; JWH-073 5OH indole: JWH-073-5-hydroxyindole; JWH-073 COOH: JWH-073 N-pentanoic acid; JWH-073: (1-butyl-1H-indol-3-yl)-1-naphthalenyl-methanone; JWH-081 5OH pentyl: JWH-081-5-hydroxypentyl; JWH-081: (4-methoxy-1-naphthalenyl)(1-pentyl-1H-indol-3-yl)-methanone; JWH-098: (4-methoxy-1-naphthalenyl)(2-methyl-1-pentyl-1H-indol-3-yl)-methanone; JWH-122: (4-methyl-1-naph-thalenyl)(1-pentyl-1H-indol-3-yl)methanone; JWH-147: (1-hexyl-5-phenyl-1H-pyrrol-3-yl)-1-naphthalenyl-methanone; JWH-200: [1-(2-morpholin-4-ylethyl)indol-3-yl]-naph- thalen-1-ylmethanone; JWH-201: 2-(4-methoxyphenyl)-1-(1-pentyl-1H-indol-3-yl)-ethanone; JWH-250 5OH pentyl: JWH-250-5-hydroxypentyl; JWH-250: 1-(1-pentyl-1H- indol-3-yl)-2-(2-methoxyphenyl)-ethanone; JWH-251: 2-(2-methylphenyl)-1-(1-pentyl-1H-indol-3-yl)-ethanone; JWH-307: [5-(2-fluorophenyl)-1-pentyl-1H-pyrrol-3-yl]-1-naphthalenyl-methanone; JWH-398: (4-chloro-1-naphthalenyl)(1-pentyl-1H-indol-3-yl)-methanone; LC: liquid chromatography; LD: liquid desortion; MAM-2201 COOH: MAM-2201 N-pentanoic acid; MAM-2201: [1-(5-fluoropentyl)-1H-indol-3-yl](4-methyl-1-naphthalenyl)-methanone; MBDB: methylbenzodioxolylbutanamine; MBPP: methylbenzylpiperazine; mCPP: 1-(3-chlorophenyl) piperazine; MD: methedrone; MDAI: 5,6-methylenedioxy-2-aminoindane; MDPBP: 3′4′-methylenedioxy-α-dimethylamino-isovalerophenone; MDPPP: 3′,4′-methylenedioxy-α-pyrrolidinopropiophenone; MDPV: methylenedioxy-pyrovalerone; MeOH: methanol; MEOPP: 1-(4-methoxyphenyl) piperazine; MEPE: methylephedrine; MEPS: microextraction by packed sorbent; MET: methoxetamine; MIP: molecularly imprinted polymer; ML: methylone; MP: monolithic polymer; MPD: mephedrone; MPHP: 4′-methyl-α-pyrrolidinohexiophenone; MS/MS: tandem mass spectrometry; MS: mass spectrometry; MT-45: 1-cyclohexyl-4-(1,2-diphenylethyl)-piperazine; MXD: mexedrone; NaCl: sodium chloride; NBPN: norbuprenorphine; n-C7: n-heptane; NH_3_: ammonia; NH4OH: ammonium hydroxide; NM2AI: N-methyl-2- aminoindane; NM-2-AI: N-methyl-2-aminoindane; NPR: naphyrone; NVP: N-vinylpyrrolidone; PALME: parallel artificial liquid membrane extraction; PD: pentedrone; PDMS: polydimethylsiloxane; PL: pentylone; PLE: pressurized liquid extraction; PPP: 1-piperonylpiperazine; PTL: pentylon; PV: pyrovalerone; RCS4: (4-methoxyphenyl)(1-pentyl-1H-indol-3-yl)methanone; SCX: silica-based cationic exchange polymer; TFMPP: 1-(3-trifluoromethylphenyl) piperazine; TOA: trioctylamine; UHPLC: ultra-high-performance liquid chromatography; UPLC: ultra-performance liquid chromatography; UR-144 4OH pentyl: UR-144-N-(4-hydroxypentyl); UR-144: ((1-pentyl-1H-indol-3-yl)(2,2,3,3-tetramethylcyclopropyl)methanone); US: ultrasound; WIN-55: [(3S)-2,3-dihydro-5-methyl-3-(4-morpholinylmethyl)pyrrolo[1,2,3-de]-1,4-benzoxazin-6-yl]-1-naphthalenyl-methanone, methanesulfonate; XLR-11 4OH pentyl: XLR-11 N-(4-hydroxypentyl); XLR11: (1-(5-fluoropentyl)-1H-indol-3-yl)(2,2,3,3-tetramethylcyclopropyl)methanone; α-PHP: α-pyrrolidinohexiophenone; α-PVP: α-pyrrolidinopentiophenone.

**Table 8:** Comparison of the main characteristics of the top-eight most frequently applied microextraction-based techniques for screening-controlled drugs in the forensic context.

		Microextraction-Based Techniques							
		Sorbent-Phase					Liquid-Phase		
**Analytical** **Framework**	Characteristics	SPME	SBSE	BAµE	MEPS	µSPE	DLLME	HP-LPME	SDME
**Sample** **Preparation**	User-friendly	++	+++	+++	+	+	++	+	+
	Eco-friendly	+++	++	++	+	+	++	+	++
	Reusability	++	+++	+	+	+	−	++	−
	Cost-effective	+	+	+++	++	++	+++	++	+++
	Routine work	+++	++	++	+	+	+	+	+
**Instrumental ** **Systems**	Online coupling	+++	++	++	+	+	+	−	+
	Comprehensive	++	++	+++	+	+++	+	++	+
**Performance**	Enrichment factor	+++	+++	++	+	+	+	++	++
	Recovery yields	+	++	++	++	++	+	+	+
	Precision level	++	++	++	+	+	+	++	+

The “−“ signal means that the technique is unsuitable for that particular characteristic. The “+”, “++”, and “+++” signals means that the microextraction-based approaches are suitable, very suitable, or extremely suitable for that particular characteristic, respectively.

## Data Availability

Not applicable.

## Supplementary Materials

The following are available online, Table S1: Microextraction-based approaches for the analysis of opioids and related substances in the forensic matrix, Table S2: Microextraction-based approaches for the analysis of cocaine and related substances in the forensic matrix, Table S3: Microextraction-based approaches for the analysis of amphetamine and related substances in the forensic matrix, Table S4: Microextraction-based approaches for the analysis of cannabinoids and related substances in the forensic matrix, Table S5: Microextraction-based approaches for the analysis of dissociative drugs and related substances in the forensic matrix, Table S6: Microextraction-based approaches for the analysis of new psychoactive substances (NPS) and related substances in the forensic matrix.
