# Supplementary material for: Application of Microextraction-Based Techniques for Screening-Controlled Drugs in Forensic Context—A Review
Source: Molecules. 2021 Apr 9;26(8):2168. doi: 10.3390/molecules26082168 (PMC8070059; doi:10.3390/molecules26082168)
Supplement: Supplementary file 1 [file molecules-26-02168-s001.pdf]

**Table S1 - Opioids**

| **Drugs** | Matrices | Sample amount | Sample pre-treatment | Microextraction technique | Optimized experimental conditions | Instrumental system | LOD (μg/L) | Absolute recovery (%) | Precision (%) | Ref |
|---|---|---|---|---|---|---|---|---|---|---|
| **MTD** | Urine | 20 mL | Not needed | DI-SPME | • Sorbent: Cu nanocomposites<br>• Extraction: 20 min (pH 9)<br>• LD: ACN (0.4 mL, 5 min) | HPLC-UV | 0.2 | n.a. | ≤ 8.96 | [44] |
| **MTD** | Urine<br>Whole blood<br>Plasma | 0.75 mL | • Dilution<br>• pH adjustment<br>• Protein precipitation<br>• Centrifugation | LV-ESSM-LLME | • Extraction: 10 min (pH 4, 12 V, 1000 rpm)<br>• SLM: NPOE<br>• AP: HCl 10 mmol/L (0.025 mL) | HPLC-UV | 0.7-2.0 | 23-28 | ≤ 7.5 | [45] |
| | | 1.5 mL | | LV-EME | • Extraction: 20 min (pH 4, 15 V, 700 rpm)<br>• SLM: NPOE<br>• AP: HCl 10 mmol/L (0.025mL) | | 1.0-2.5 | 57-69 | ≤ 7.0 | |
| **MTD** | Urine<br>Plasma | 10 mL | • pH adjustment<br>• Dilution<br>• Protein precipitation<br>• Centrifugation | DES-AAELLME | • Extraction: 100 μL of TNO:CC (1:2 molar ratio) at pH 10 + 100 μL THF<br>• Pushing and pulling from a syringe × 10<br>• Centrifugation<br>• Supernatant injected (1 μL) | GC-FID | 0.7 | 97.4 | ≤ 5.4 | [46] |
| **MTD** | Plasma | 0.2 mL | - | μSPE | • Sorbent: MIP<br>• Extraction: 10 min by shaking (pH 7.4, 3 % NaCl)<br>• LD: MeOH (0.2 mL, 6 min) | LC-MS/MS | 1 | 80 | ≤ 8.0 | [47] |
| **MTD** | Urine | 10 mL | • pH adjustment<br>• Lipidic precipitation<br>• Centrifugation | MSPE | • Sorbent: MNGO<br>• Extraction: 15 min (1100 rpm, pH 10)<br>• LD: ACN (0.4 mL, 5 min) | HPLC-UV | 25 | 97 | ≤ 7.98 | [48] |
| **MTD** | Urine | 2 mL | • Centrifugation<br>• Dilution | DLLME-SFO | • Sample: NaCl (1.3% w/v) and borate buffer (pH 8.9)<br>• DS: MeOH (580 μL)<br>• ES: 1-undecanol (58 μL)<br>• Rapid injection<br>• Centrifugation<br>• SFOs collected and injected. | HPLC-UV | 1.67 | 97 | ≤ 5.12 | [49] |
| | Serum | 1 mL | • Protein denaturation and precipitation<br>• Centrifugation<br>• Dilution | | | | 3.34 | 95 | | |

| | | | | | | | | | | |
|---|---|---|---|---|---|---|---|---|---|---|
| **MTD** | EBC | 1 mL | Dilution | DLLME and ULLME | DLLME<br>• Sample: pH 10<br>• DS: MeOH (1 mL)<br>• ES: $CHCl_3$ (200 µL)<br>• Rapid injection<br>• Centrifugation<br>• Evaporation on supernatant<br>• Redissolution and injection | HPLC-UV | 0.5 (LLOQ) | n.a. | ≤ 4.86 | [50] |
| | | | | | ULLME<br>• Sample: pH 10<br>• ES: $CHCl_3$ (200 µL)<br>• Sonication<br>• Centrifugation<br>• Evaporation of supernatant<br>• Redissolution and injection | | | | | |
| **MTD** | Urine | 0.1 mL | - | MEPS | • Sorbent: C8<br>• Activation: 100 µL MeOH × 3<br>• Conditioning: 100 µL $H_2O$ × 3<br>• Samples aspirated and discarded × 5<br>• Washing: 100 µL $H_2O$ x 2 + 100 µL of 5 % MeOH × 1<br>• Elution: 50 µL of 0.1% COOH in MeOH | MS/MS | 1.5 | 91.7-106.7 | ≤ 11.1 | [40] |
| **MTD** | Plasma | 4 mL | • Protein precipitation<br>• Centrifugation<br>• Dilution | SBME | • Extraction: pH 11.5 with 5% NaCl, 20 ºC, 700 rpm, 45 min<br>• ES: 1-undecanol (25 µL) | GC-FID | 7 | 15 | ≤ 7.3 | [51] |
| | Urine | 10 mL | Not needed | | | | 5.5 | 15.8 | | |
| **MTD** | Urine | 3 mL | Not needed | TF-SPME | • Coating: C18- polyacrylonitrile<br>• Precondition: 30 min in MeOH/$H_2O$ (1:1)<br>• Extraction: 10 min, 1200 rpm | DART-MS/MS | > 0.5 | n.a. | ≤ 4 | [52] |

| | | | | | | | | | | |
|---|---|---|---|---|---|---|---|---|---|---|
| **MTD** | Plasma | 50 µL | Centrifugation | MEPS | • Sorbent: C18<br>• Activation: 100 µL ACN × 3<br>• Conditioning: 100 µL $H_2O$ × 3<br>• Samples aspirated and discarded × 15<br>• Washing: 100 µL $H_2O$ × 1 + 100 µL 5 % ACN × 1<br>• Elution: 250 µL ACN × 2<br>• Evaporation of supernatant<br>• Redissolution and injection | HPLC-CD | 1.2 | 90.1-95.5 | ≤ 5.8 | [53] |
| | Dried blood spot | 5- 50 µL | • Solid-liquid extraction<br>• Evaporation to dryness<br>• Redissolution | | | | | | | |
| **MTD** | Urine | Urine and plasma: 500 µL | Urine and plasma:<br>• Centrifugation<br>• Filtration<br>• Dilution | DLLME | • Sample at pH 10<br>• DS: MeOH (2.5 mL)<br>• ES: $CHCl_3$ (200 µL)<br>• Centrifugation<br>• Sediment dried and redissolved | HPLC-UV | 4.9 | 98.6-100.3 | ≤ 6.4 | [42] |
| | Plasma | | | | | | 7.3 | | | |
| | Saliva | Saliva and sweat: 100 µL | Saliva and sweat: Dilution | | | | 25.12 | | | |
| | Sweat | | | | | | 24.85 | | | |
| **MTD** | Urine<br>Plasma | 5 mL | • Salt addition<br>• pH adjustment | HS-SPME | • Coating: PPy-α- COOH<br>• Extraction: 25 min, 600 rpm, pH 11, 20% NaCl, 45 ºC<br>• TD: 280ºC, 2 min | GC-FID | 0.035 | n.a. | ≤ 10.9 | [54] |

| | | | | | | | | | |
|---|---|---|---|---|---|---|---|---|---|
| **MTD** | Urine | 1.75 mL | • Dilution | HF-LPME-BE | • Extraction: 27 min (pH 9.8, 1000 rpm)<br>• BE: 9.5 min<br>• SLM: dibutyl ether (80 µL)<br>• AP: phosphate buffer (0.01 M), 7 µL, pH 2.7 | HPLC-UV | 0.2 | 77 | ≤ 5.9 | [55] |
| | Plasma | 1.16 mL | | | | | | | | |
| **MTD** | Urine | 50 µL | Samples used without pre-treatment | MEPS | • Sorbent: C8<br>• Activation: 50 µL MeOH × 1<br>• Conditioning: 50 µL $H_2O$ × 1<br>• Samples aspirated and discarded × 5<br>• Elution: 30 µL MeOH × 1, 40 ºC, 2 min | GC-MS | 0.4 | 60 | ≤ 15 | [56] |
| **EDDP MTD** | Saliva | 0.1 mL | Dilution | DI-SPME | • Coating: PDMS<br>• Extraction: 30 min<br>• TD: 5 min, 250ºC | GC-MS | 4-8 | 97.9 -103.6 | ≤ 4.3 | [57] |
| **EDDP MTD** | Hair | 50 mg | • Hydrolysis<br>• Dilution<br>• Salt addition<br>• pH adjustment | DI-SPME | • Coating: PDMS<br>• Extraction: 30 min, pH 9.2, 10% NaCl<br>• TD: 5 min, 250ºC | GC-MS | 0.15-2.48 ng/mg | 103.6-107.2 | ≤ 13.3 | [58] |
| **EDDP MTD** | Plasma | 0.1 mL | • Dilution<br>• pH adjustment | DI-SPME | • Coating: PDMS<br>• Extraction: 30 min, pH 9<br>• TD: 10 min, 250ºC | GC-MS | 5-9 | n.a. | < 5.2 | [59] |
| **EDDP EMDP MTD** | Hair | 10 mg | Digestion | HS-SPME | • Coating: PDMS/DVB<br>• Extraction: 20 min, 110ºC, 150 rpm<br>• TD: 5 min, 260ºC | GC-MS | 0.03-0.05 ng/g | 10.5-17.4 | ≤ 7.2 | [60] |
| **MTD PTD** | Urine | 3 mL | • pH adjustment<br>• Dilution<br>• Salt addition | SPME | • Coating: PDMS<br>• Extraction: 30 min, 15% NaCl, pH 11<br>• TD: 240ºC, 1 min | GC-NPD | < 1 | n.a. | ≤ 8.7 | [61] |
| **TMD** | Rabbit brain tissue | 2 g | • Solid-liquid extraction<br>• Centrifugation<br>• Evaporation<br>• Dilution | DI-SPME | • Washing:1 mL acetone:ACN (3:1, v/v)<br>• Eluting step:0.2 mL HCl (1 mol/L):MeOH (1:1, v/v) | HPLC-UV | 1 | 76.2-91.2 | ≤ 8.2 | [43] |
| **TMD** | Urine | 7 mL | • pH adjustment<br>• Salt addition<br>• Dilution | DI-SPME | • Support: MWCNTf-ZnO<br>• Solvent: 1-octanol (1.5 min)<br>• Extraction: 30 min (800 rpm), 30% NaCl, pH 11<br>• LD: MeOH (50 µL), 15 min | GC-FID | 0.03 | n.a. | ≤ 5.43 | [62] |

| | | | | | | | | | | |
|---|---|---|---|---|---|---|---|---|---|---|
| **TMD** | Urine<br>Blood | Not available | • Dilution<br>• pH adjustment | DLLME | • Sample at pH 12<br>• DS: EtOH (1.0 mL)<br>• ES: $CCl_4$ (30 µL)<br>• Centrifugation (5000 rpm, 3 min) | GC-MS | 0.08 | n.a. | ≤ 3.6 | [63] |
| **TMD** | Urine | 9 mL | • pH adjustment<br>• Salt addition | SA-DSDME | • Sample pH at 10.7, 5% NaCl with Triton X-100 (36 µL, 50 mmol $L^{-1}$).<br>• ES: *n*-octanol (14 µL)<br>• Extraction: 25 min at 55ºC (750 rpm) | GC-FID | 5.1 | 63.8 | ≤ 10 | [64] |
| | Plasma | 4 mL | • Protein precipitation<br>• Centrifugation<br>• Dilution<br>• pH adjustment<br>• Salt addition | | | | 6.5 | 60.8 | | |
| **TMD** | Urine | 5 mL | • pH adjustment<br>• Salt addition<br>• Centrifugation | BS-DLLME | • Sample at pH 12, 7.5% NaCl;<br>• DS: acetone (0.6 mL)<br>• ES: ethyl acetate (30 µL) and $CHCl_3$ (70 µL)<br>• Centrifugation<br>• Sediment collected and dried<br>• Redissolution | HPLC-FD | 0.2 | n.a. | ≤ 4.1 | [65] |
| **TMD** | Urine | 1 mL | • pH adjustment<br>• Dilution<br>• Filtration | PFSPE | • Sorbent: PMMA/PS<br>• Conditioning: EtOH (100 µL) and $H_2O$ (100 µL)<br>• Extraction: Sample pushed through the syringe<br>• Elution: MeOH (200 µL) | CD-IMS | 1.6 | n.a. | ≤ 9.5 | [66] |
| | Plasma | 1.5 mL | • Protein precipitation<br>• Centrifugation<br>• Filtration<br>• pH adjustment<br>• Dilution | | | | 9.4 | n.a. | | |
| **TMD** | Urine | 12 mL | Urine samples used without pre-treatment | SBME | • Extraction: pH 12.0, 1000 rpm, 25 min<br>• ES: *n*-nonanol (4 µL) | GC-MS | 0.02 | 86-109 | ≤ 5.9 | [67] |
| | Plasma | 2 mL | Dilution | | | | | | | |

| | | | | | | | | | |
|---|---|---|---|---|---|---|---|---|---|
| **TMD** | Urine<br>Plasma | 5 mL | • Protein precipitation<br>• Dilution<br>• pH adjustment | HF-LPME | • Extraction: 40 min (pH 11, 4 mol/L NaCl, 1000 rpm)<br>• SLM: *n*-C12<br>• AP: ACN (25 µL) | GC-MS | 0.08 | n.a. | ≤ 7.9 | [68] |
| **TMD** | Urine | 2 mL | pH adjustment | LPME | • Sample at 0.01 M NaOH, pH 11.5<br>• Extraction: *n*-C8 (100 µL), 25 min, 1250 rpm, 50ºC<br>• BE: 3.5 µL phosphate buffer (0.01 mol/L, pH 2.5), 25 min, 1250 rpm, 50ºC | HPLC-FD | 0.12 | 64 | ≤ 6.29 | [69] |
| | Plasma | 0.4 mL | • pH adjustment<br>• Dilution | | | | | | | |
| **TMD** | Plasma | 0.5 mL | • pH adjustment<br>• Dilution | SPME | • Coating: PDMS/DVB<br>• Extraction: 30 min, 100 ºC, 2000 rpm<br>• TD: 2 min, 250ºC | GC-MS | 0.2 | n.a. | ≤ 7.8 | [70] |
| **MOR** | Urine | 2 mL | • pH adjustment<br>• Dilution | EME | • Extraction: 24 min (pH 6, 90 V, 1000 rpm)<br>• SLM: NPOE, with 10% TEHP and 10% DEHP<br>• AP: HCl 0.1 mol/L (20 µL, pH 1.0) | DPV(SPCE) | 1.5 | 71-76 | ≤ 9.4 | [71] |
| **MOR** | Hair | 50 mg | • Washing<br>• Digestion<br>• Solid-liquid extraction<br>• Filtration<br>• Dilution | MSPE | • Sorbent: SMMNP<br>• Extraction: 20 min (1100 rpm, pH 9)<br>• LD: MeOH (0.3 mL, 10 min) | HPLC-UV | 0.1 | n.a. | ≤ 2.59 | [72] |
| **COD**<br>**MOR** | Urine | 2 mL | • Filtration<br>• Sonication<br>• Dilution | BAµE | • Coating: AC<br>• Extraction: 2.5 h, 1000 rpm (pH 7)<br>• LD: MeOH/ACN (1:1, 1.5 mL), 30 min<br>• Evaporation to dryness and redissolution | HPLC-DAD | 0.06-0.90 | 38.4-41.3 | ≤ 8.0 | [41] |
| **6-MAM**<br>**COD**<br>**MOR** | Hair | 10 mg | • Digestion<br>• Evaporation<br>• Derivatization | HS-SPME | • Coating: PDMS<br>• Extraction: 25 min, 125 ºC<br>• TD: 10 min, 260ºC | GC-MS | 2-5 µg/g | n.a. | ≤ 14.8 | [73] |

| | | | | | | | | | |
|---|---|---|---|---|---|---|---|---|---|
| **COD**<br>**MOR**<br>**OXY** | Urine | 4.8 mL | • Dilution<br>• pH adjustment | EME | • Extraction: 10 min (pH 5, 90 V) 700 rpm<br>• SLM: NPOE containing 15% (v/v) DEHP<br>• AP: HCl 100 mmol/L (10 μL) | HPLC-UV | 10.0-50.0 | n.a. | ≤ 12.6 | [74] |
| **BPN**<br>**MTD**<br>**N-BPN**<br>**NLX** | Plasma | 0.1 mL | Not necessary | MEPS | • Sorbent: C8<br>• Activation: 100 μL ACN x 3<br>• Conditioning: 100 μL $H_2O$ x 3<br>• Samples aspirated and discarded x 15<br>• Washing: 100 μL $H_2O$ x 2 + 100 μL of 5% ACN x 1<br>• Elution: 250 μL ACN x 2<br>• Evaporation to dryness and reconstitution | HPLC-CD | 0.04-0.9 | 86-96 | ≤ 4.3 | [75] |
| **FTY** | Urine | 0.8 mL | Not necessary | DLLME | • Sample at pH 10;<br>• DS: 2-propanol (0.1 mL)<br>• ES: chlorobenzene (30 + 20 μL) and $CHCl_3$ (70 μL)<br>• Sonication (11 min, 45ºC)<br>• Centrifugation<br>• Sediment collected and redissolved | GC-MS | 1.0 | 88.9-102. | ≤ 14.3 | [76] |
| **FTY** | Urine<br>Plasma | 5 mL<br>0.5 or 0.25 mL | Dilution | Online-CME | • Sorbent: MIX<br>• Extraction: sampling flow rate of 7 mL/min, 25 min<br>• LD: MeOH (5 mL, 3 min) | HPLC-UV | 3 | n.a. | ≤ 12 | [77] |
| **FTY** | Breath | 18 mL | • Gas-liquid extraction<br>• Evaporation<br>• Reconstitution | DI-SPME | • Sorbent: PDMS/DVB<br>• Extraction: 60 min, 85ºC<br>• TD: 265ºC, 5 min | GC-MS | 10 | n.a. | ≤ 12.1 | [78] |
| **FTY** | Urine | 1.8 mL | • pH adjustment<br>• Dilution | SDME | • Sample at 0.01 mol/L NaOH, pH at 10.7<br>• Extraction: *n*-C8 (100 μL), 30 min, 1000 rpm, 30 ºC | HPLC-UV | 0.1 | 49 | ≤ 8.8 | [79] |

| | | | | | | | | | | |
|---|---|---|---|---|---|---|---|---|---|---|
| | Plasma | 0.75 mL | | | • BE: 5 µL percloric acid (0.001 mol/L), 20 min, 700 rpm, 30 ºC | | | | | |
| **FTY** | Plasma | 1 mL | • Protein precipitation<br>• Centrifugation<br>• pH adjustment<br>• Salt addition | HS-SPME | • Coating: PDMS<br>• Extraction: 30 min, pH 11, 25% NaCl and 25 % $K_2CO_3$, 80ºC<br>• TD: 270ºC, 5 min | GC-MS | 0.03 | n.a. | ≤ 4.9 | [80] |
| **FTY** | Plasma | 1 mL | • Dilution<br>• pH adjustment<br>• Salt adittion | DI-SPME | • Coating: PDMS/DVB<br>• Extraction: 30 min, 6% NaCl<br>• Washing: $H_2O$ 30s, 20% MeOH 30 s<br>• TD: 270ºC, 5 min | GC-MS | 0.8<br>(LOQ) | n.a. | ≤ 7.0 | [81] |
| **AFT**<br>**FTY**<br>**SFT** | Urine | 2.5 mL | Both:<br>• Filtration<br><br>DLLME:<br>• Dilution | DLLME<br>HF-LPME | DLLME<br>• Sample at pH 10;<br>• DS: MeOH (2 mL)<br>• ES: $CHCl_3$ (162 µL)<br>• Shaking (10 s)<br>• Centrifugation (3500 rpm, 5 min)<br>• Sediment collected and redissolved<br><br>HF-LPME<br>• Extraction: 20 min (45ºC, 0.5 mol/L NaOH, 1200 rpm)<br>• SLM: isoamyl benzoate<br>• AP: $H_2SO_4$ solution (0.05 mol/L, 10 µL) | HPLC-DAD | DLLME:<br>0.4-1.9<br><br>HF-LPME:<br>1.1-2.3 | n.a. | ≤ 15.9 | [82] |
| | Plasma | 1.25 mL | Both:<br>• Filtration<br>• Dilution<br><br>DLLME:<br>• Protein precipitation<br>• Centrifugation<br>• pH adjustment | | | | | | | |
| **COD** | Urine | Not available | • Filtration<br>• Dilution | TFME | • Conditioning: 5 min using 20 mmol/L Tris-HCl, 140 mmo/L NaCl, 5 mmol/L KCl, and 1 mmol/L $MgCl_2$ at pH 7.4 solution and allowed to dry<br>• Extraction: 30 min (400 rpm, 25ºC)<br>• LD: 200 µL (ACN/ $H_2O$, 90/10, v/v) under vortex (10 min)<br>• Evaporation to dryness and redissolution | EI-IMS | 3.4 | 90.1 | ≤ 8.1 | [83] |

| | | | | | | | | | | |
|---|---|---|---|---|---|---|---|---|---|---|
| **HER<br>OXYC** | Urine | 0.25 mL | Filtration | Online-CME | • Sorbent: C18 or C18-Phe<br>• Extraction: sampling flow rate of 0.02 mL/min, 12.5 min<br>• LD: MeOH (0.25 mL, 2.5 min) | MS | 0.6-10.9 | n.a. | ≤ 5.9 | [84] |
| **MOR** | Urine | Not available | pH adjustment | DI-SPME | • Coating: Peptide functionalized CNTs<br>• Extraction: 5 min sonication, pH 7 (40ºC)<br>• LD: $H_2O$/MeOH (80/20, v/v, 200 µL), 2 min | HPLC-UV | 0.1 | n.a. | n.a. | [85] |
| **COD<br>MOR<br>MTD** | Urine | 2 mL | • Centrifugation<br>• Filtration<br>• Dilution<br>• pH adjustment | DLLME | • Sample at pH 9.8;<br>• DS: acetone (0.5 mL)<br>• ES: $CHCl_3$ (300 µL)<br>• Shaking (30 s)<br>• Centrifugation (10 min, 4000 rpm)<br>• Sediment collected and redissolved | HPLC-DAD | 9-25 | 87.5-107.9 | ≤ 6.1 | [86] |
| **MOR** | Urine | 1 mL | Not available | DI-SPME | • Coating: CNTs<br>• Extraction: 2 min electric field (1 V); pH 5 (45ºC)<br>• LD: $H_2O$/MeOH (80/20, v/v, 200 µL) using an electric field (1 V) | HPLC-UV | 0.15 | 77 | n.a. | [87] |
| **COD<br>MOR** | Urine | 10 mL | • Centrifugation<br>• Filtration<br>• Dilution.<br>• pH adjustment<br>• Salt adittion | DLLME-SFO | • Sample: NaCl (1.3% w/v) and $K_2CO_3$ (10% w/v, pH 10.2)<br>• DS: MeOH (150 µL)<br>• ES: 1-undecanol (40 µL)<br>• Centrifugation (5000 rpm, 5 min)<br>• Ice bath and extraction (5 min)<br>• SFOs solidified, collected and injected<br>• Rapid injection | HPLC-UV | 5.0-17.0 | n.a. | ≤ 4.6 | [88] |
| | Hair | 20 mg | • Washing<br>• Digestion<br>• Filtration<br>• Evaporation<br>• Dilution<br>• pH adjustment | | | | 5.0-8.5 µg/g | | | |

| | | | | | | | | | | |
|---|---|---|---|---|---|---|---|---|---|---|
| **MTD** | Sweat | Cotton pad (0.5 × 0.5 cm) 4-6 rubbing on the forehead | • Acidic extraction<br>• pH adjustment | HS-SPME | • Coating: PDMS<br>• Extraction: 10 min (90ºC) + 3 min (90ºC) for derivatization<br>• TD: 3 min, 250ºC | GC-MS | 0.08 ng/pad | 102.8 | ≤ 9.9 | [89] |
| **AFT**<br>**BPN**<br>**EDDP**<br>**FTY**<br>**MOR**<br>**MTD**<br>**NBP**<br>**NFT**<br>**6-MAM**<br>**SFT** | Blood | 0.5 mL | • Protein precipitation<br>• Centrifugation<br>• Dilution<br>• Salt addition<br>• pH adjustment | DLLME | • DS: 0.25 mL of MeOH<br>• ES: 100 µL $CHCl_3$<br>• Sonication (1 min)<br>• Centrifugation (4000 rpm,5 min)<br>• Infranatant collected, evaporated and redissolved<br>• Rapid injection | UPLC-MS/MS | 0.05-2 | 3-117 | ≤ 15 | [90] |
| **6-MAM**<br>**BPN**<br>**COD**<br>**DAM**<br>**EDDP**<br>**MOR**<br>**MTD**<br>**NBP** | Oral fluid | 0.12 mL | • Dilution<br>• pH adjustment<br>• Protein precipitation<br>• Sonication<br>• Centrifugation | MEPS | • Sorbent: C18<br>• Conditioning: 250 µL MeOH × 2 + 250 µL $H_2O$/MeOH (80:20, v/v) × 2<br>• Samples aspirated and discarded × 5<br>• Washing: 100 µL 50 mM $NH_3$ in $H_2O$/MeOH (90:10, v/v) × 3<br>• Elution: 100 µL 5 mM COOH in MeOH × 5 | LC-MS/MS | 0.2-2 | 67-102 | ≤ 20 | [91] |
| **MTD**<br>**PTD** | Blood | 1 mL | • Dilution<br>pH adjustment | DLLME<br>HF-LPME | DLLME<br>• ES and DS: 100 µL toluene<br>• Sonication (3 min) and manual shaking<br>• Centrifugation (10000 rpm,3 min)<br>• Supernatant collected<br><br>HF-LPME<br>• SLM: toluene<br>• AP: toluene (10 µL) | GC-MS | 0.5-2.5 | DLLME:<br>83.3-99.1<br><br>HF-LPME<br>79.4-92.7 | DLLME:<br>≤ 5.2<br><br>HF-LPME<br>≤ 3.7 | [92] |

| | | | | | | | | | | |
|---|---|---|---|---|---|---|---|---|---|---|
| | Urine | | | | • Extraction: 10 min (500 rpm, pH 13.0, 30ºC)<br>• For blood samples, 10 mg of NaCl was added to break emulsion | | | | | |
| **6-MAM**<br>**BPN**<br>**COD**<br>**DMP**<br>**EDDP**<br>**EMOR**<br>**HER**<br>**MTD**<br>**NPD**<br>**PTD** | Urine | 4 mL | • Centrifugation<br>• Filtration<br>• pH adjustment | DLLME | • DS: 1400 μL isopropanol<br>• ES: 600 μL DCM<br>• Extraction: pH ≥ 11.5<br>• Rapid injection<br>• Centrifugation (10000 rpm,5 min)<br>• Infranatant collected, evaporated and redissolved | CE-TOF-MS | 0.25-10 | 0-107 | n.a. | [93] |
| **COC**<br>**MOR** | Horse urine | 10 mL | pH adjustment | SDME | • Solvent: 5 μL of $CHCl_3$/MeOH (90:10, v:v)<br>• Extraction: 20 min (25ºC, pH 11, 500 rpm)<br>• Evaporation and redissolution | OT-CEC | 5.12-8.27 | 103.1-108.3 | ≤ 2.1 | [94] |
| **ACOD**<br>**COD**<br>**HER** | Urine | 2 mL | • Filtration<br>• pH adjustment<br>• Dilution | MSPE | • Sorbent: $Fe_3O_4$/$SiO_2$/poly(MAA-*co*-EDMA)<br>• Extraction: vortex (5 s, pH 9.2)<br>• Washing: 0.5 mL $H_2O$ × 5<br>• LD: 0.04 mL of acetone containing 0.5% COOH (v/v) with 10 s vortex. | CE-DAD | 15-53 | 85.4-109.7 | ≤ 12.4 | [95] |
| **MTD** | Hair | 10 mg | • Washing<br>• Drying<br>• Digestion<br>• Salt addition | HS-SPME | • Coating: PDMS<br>• Extraction: 10 min (90 ºC)<br>• Derivatization (3 min, 90ºC, with 5 μL of acetic anhydride)<br>• TD: 4 min, 260ºC | GC-MS | 0.05 ng/mg | 9.5 | ≤ 9.9 | [96] |
| **COD**<br>**MTD**<br>**OXYC**<br>**MFN,**<br>**BZC,**<br>**LDC,**<br>**PRC,** | Urine | 0.5 mL | • Dilution<br>• pH adjustment<br>• Salt addition | DI-SPME | • Coating: PDMS/DVB<br>• Extraction: 30 min (pH 12, 40 ºC)<br>• Derivatization<br>• TD: 260ºC, 2 min | GC-MS | 20-200 | 90-114 | ≤ 10.9 | [97] |

| | | | | | | | | | | |
|---|---|---|---|---|---|---|---|---|---|---|
| **MPV, TTC, BPC, DBC, PTC** | | | | | | | | | | |
| **MTD** | Hair | 20 mg | • Washing<br>• Drying<br>• Digestion<br>• pH adjustment<br>• Salt addition | HS-SPME | • Coating: PDMS<br>• Extraction: 5 min (90ºC, pH 10.3)<br>• TD: 25 ºC, 3 min | GC-MS | 0.35 ng/mg | n.a. | ≤ 7.4 | [98] |
| **MOR NLX MTD 6-MAM** | Oral fluid | 0.5 mL | • Dilution<br>• Salt addition<br>• Protein precipitation | US-DLLME | • DS: 1.4 mL MeOH<br>• ES: 200 µL $CHCl_3$<br>• Extraction: 2.5% NaCl, pH 8.0<br>• Ultrassound for 5 min<br>• Centrifugation (3500 rpm, 5 min)<br>• Infranatant collected, evaporated and redissolved | UPLC-MS/MS | 0.1-25 | 80.9-129 | ≤ 14.5 | [99] |
| **MOR COD 6-MAM** | Hair | 10 mg | • Washing<br>• Digestion<br>• PLE extraction<br>• Evaporation<br>• Redissolution<br>• Centrifugation<br>• Dilution<br>• pH adjustment<br>• Salt addition | PLE-DLLME | • DS: 500 µL of 2-propanol<br>• ES: 200 µL of $CHCl_3$<br>• Extraction: 24% NaCl, pH 11.0, 10% iso-propanol<br>• Sonication for 10 min<br>• Centrifugation (9000 rpm, 5 min, 3ºC)<br>• Infranatant collected, evaporated and redissolved | HPLC-HRMS/MS | 0.2-0.5 pg/mg | 33-49 | ≤ 21 | [100] |
| **COD MOR** | Urine and plasma | 7 mL | • Dilution<br>• pH adjustment | EME | • Extraction: 30 min (pH 6, 25 V) 500 rpm<br>• SLM: Agarose gel 1 % (m/v) pH 3.0, 15 m thickness<br>• AP: same as SLM | HPLC-UV | 1.5 | 68-74 | ≤ 10.3 | [101] |
| **TMD MTD** | Urine, plasma and saliva | 2 mL | • Dilution<br>• pH adjustment<br>• Filtration<br>• Centrifugation | MSPE | • Sorbent: Zn-Al layered double hydroxide intercalated with tyrosine<br>• Extraction: ultrasound (5 min, pH 8)<br>• LD: 0.1 mL of acetone, 15 min under sonication | GC-MS | 0.15-2.5 | n.a. | ≤ 10.7 | [102] |
| **MTD** | Saliva | 1 mL | • Dilution<br>• pH adjustment<br>• Filtration | US-DLLME | • ES: 1 mL $CHCl_3$<br>• Extraction: pH 8.0<br>• Ultrasound for 5 min<br>• Centrifugation (3500 rpm, 5 min)<br>• Infranatant collected, evaporated and redissolved | GC-MS | 50.0 | 94.2-107.5 | ≤ 7.9 | [103] |
| **MOR 6-MAM** | Urine | 1 mL | pH adjustment | MSPE | • Sorbent: Graphene oxide – $Fe_3O_4$<br>• Extraction: vortex (15 min, pH 6) | UHPLC-MS/MS | 0.02-0.03 | 82.7-89.4 | ≤ 10.6 | [104] |

| | | | | | | | | | |
|---|---|---|---|---|---|---|---|---|---|
| **COD** | | | | | • LD: 3 mL of MeOH with 10 % ammonia, 10 min vortex | | | | |
| **TMD** | Urine | 1.5 mL | • Dilution<br>• pH adjustment | EME | • Extraction: 20 min (pH 4, 180 V) 1000 rpm<br>• SLM: Porous Aromatic Framework-48 with the nitro functional groups with NPOE<br>• AP: 100 mmol/L HCl (20 μL) | IMS | 1.5 | 94-98 | ≤ 4.7 | [105] |

**Table S2 - Cocaine**

| **Drugs** | Matrices | Sample amount | Sample pre-treatment | Microextraction technique | Optimized experimental conditions | Instrumental system | LOD (µg/L) | Absolute recovery (%) | Precision (%) | Ref |
|---|---|---|---|---|---|---|---|---|---|---|
| **BE**<br>**BN**<br>**CCE**<br>**COC**<br>**ECG**<br>**m-HOBE**<br>**NC**<br>**NCE** | Urine | 1 mL | • Dilution<br>• pH adjustment<br>• Centrifugation | MDSPE | • Sorbent: DVB+VP@SMPS@$Fe_3O_4$<br>• Extraction: sonication (1 min) + stirring (300 rpm, 20 min)<br>• LD: 10 min (1 mL, MeOH/ACN, 4:1, v/v) | LC-MS | 0.09-1.10 | 75.1-105.7 | ≤ 6.6 | [114] |
| **COC**<br>**ECGME** | Saliva | 0.5 mL | • pH adjustment | LOV-µSPE | • Sorbent: Mixed-mode cationic/reversed-phase<br>• Washing: $H_2O$ (0.5 mL) + IPA (0.5 mL)<br>• Drying: air (1.5 mL × 2)<br>• Elution: 0.1 mL of (3% v/v) $NH_3$ in IPA | IMS | 0.14-0.3 | n.a. | ≤ 28 | [115] |
| **AEME**<br>**BE**<br>**CCE**<br>**COC** | Hair | 50 mg | • Washing<br>• Digestion<br>• Evaporation to dryness<br>• Derivatization<br>• pH adjustment | HF-LPME | • Extraction: 10 min (pH 2400 rpm, pH 9-10)<br>• SLM: DHE<br>• AP: 0.05 mol/L HCl (50-70 µL)<br>• Evaporation<br>• Derivatization | GC-MS | 0.05-0.1 ng/mg | n.a. | ≤ 14.4 | [116] |
| **CCE**<br>**COC**<br>**NC** | Breast milk | 0.5 mL | • pH adjustment<br>• Salt adittion | HF-LPME | • Extraction: 30 min (2400 rpm, pH 9.0, 25% NaCl)<br>• SLM: 1-octanol<br>• AP: 0.4 mol/L HCl<br>• Evaporation<br>• Derivatization | GC-MS | 5-7 | 32.0-67.4 | ≤ 15.9 | [112] |
| **BE**<br>**COC**<br>**ECGME** | Urine | 0.2 mL | • Centrifugation<br>• Dilution<br>• pH adjustment | MEPS | • Sorbent: 80 % C8 + 20 % SCX<br>• Conditioning: 250 µL MeOH × 1 + 250 µL 0.1% COOH × 1<br>• Samples aspirated and discarded × 6<br>• Washing: 50 µL 0.1% COOH × 4 + drying<br>• Elution: 100 µL of 1% $NH_4OH$ in MeOH × 4<br>• Evaporation to dryness and derivatization | GC-MS | 25 (LLOQ) | 14.5-83.3 | ≤ 14.38 | [111] |
| **BE**<br>**CCE**<br>**COC**<br>**ECGME** | Plasma | 0.1-1.0 mL | • pH adjustment<br>• Dilution | µSPE | • Sorbent: MMIP<br>• Extraction: 4 min (20ºC, 100 rpm, 0.2 mL *n*-C6)<br>• Elution: 2 mL DCM/IPA/$NH_4OH$ (75:20:5, v:v:v), ultrasound irradiation (5 min)<br>• Evaporation to dryness<br>• Re-dissolved with 0.04 mL of 2mM $C_2H_7NO_2$ in MeOH | LC-MS/MS | 0.000013-0.00036 | 91-102 | ≤ 10 | [113] |

| | | | | | | | | | |
|---|---|---|---|---|---|---|---|---|---|
| **BE**<br>**CCE**<br>**COC**<br>**ECGME** | Plasma | 0.1-1.0 mL | • pH adjustment<br>Dilution | µSPE | • Sorbent: MIP<br>• Extraction: 10 min (50ºC, 150 rpm)<br>• Elution: 10 mL DCM/IPA/ $NH_4OH$ (76:20:4, v:v:v), ultrasound irradiation (8 min)<br>• Evaporation to dryness<br>• Re-dissolved with 0.1 mL of 2mM $C_2H_7NO_2$ in MeOH | LC-MS/MS | 0.061-0.87 | 94-103 | ≤ 12 | [117] |
| **BE**<br>**CCE**<br>**COC**<br>**ECGME** | Urine | 5 mL | • pH adjustment<br>Dilution | µSPE | • Sorbent: MIP<br>• Extraction: 15 min (30ºC, 200 rpm)<br>• Elution: 5 mL DCM/IPA/$NH_4O$ (72:20:8, v:v:v), ultrasound irradiation (8 min)<br>• Evaporation to dryness<br>• Redissolved with 0.05 mL of 2mM $C_2H_7NO_2$ in MeOH | LC-MS/MS | 0.049-0.5 | 89-100 | ≤ 10 | [118] |
| **CCE**<br>**LVM**<br>**NC**<br>**TCOC** | Hair | 20 mg | • Washing<br>• Digestion<br>pH adjustment | DI-SPME | • Sorbent: PDMS<br>• Extraction: 1 h, 80 ºC<br>• TD: 2 min | GC-MS | 0.1 ng/mg | n.a. | ≤ 20 | [119] |
| **COC**<br>**LIDO** | Urine | 3.5 mL | • pH adjustment<br>Dilution | HF-LPME | • Extraction: 30 min (700 rpm, pH 11.0, 20 % NaCl)<br>• SLM: *n*-C12<br>• AP: ACN (25 µL) | GC-MS | 0.01-0.05 | 25-35 | ≤ 9.3 | [120] |
| **AEME**<br>**BE**<br>**CCE**<br>**COC** | Meconium | 0.3 g | • Digestion<br>• Agitation<br>• Centrifugation<br>pH adjustment | DPX | • Sorbent: SCX<br>• Conditioning: 1 mL ACN<br>• Samples sucked and held for 1 min<br>• Washing: 1 mL $H_2O$<br>• Elution: DCM/IPA/$NH_4OH$ (78:20 :2, v/v/v), × 3, 1 mL<br>• Evaporation to dryness and derivatization | GC-MS | 2.5-15.0 ng/mg (LLOD) | 50.7-98.9 | ≤ 12.05 | [121] |
| **COC** | Urine | 3 mL | Not needed | TF-SPME | • Coating: C18- polyacrylonitrile<br>• Precondition: 30 min in MeOH/$H_2O$ (1:1)<br>• Extraction: 10 min, 1200 rpm | DART-MS/MS | > 0.5 | n.a. | ≤ 4 | [52] |
| **CCE**<br>**COC** | Plasma | 0.4 mL | • Centrifugation<br>pH adjustment | DI-SPME | • Coating: PDMS<br>• Extraction: pH 9.0, 25 min, 5 % NaCl<br>• TD: 5 min, 250 ºC | GC-MS | 11-19 | n.a. | ≤ 19.2 | [122] |
| **CCE**<br>**COC** | Hair | 50 mg | • Washing<br>• Enzymatic hydrolysis<br>• Dilution<br>pH adjustment | DI-SPME | • Coating: PDMS<br>• Extraction: pH 8.5, 20 % NaCl, 25 min<br>• TD: 5 min, 250 ºC | GC-MS | 0.02-0.08 ng /mg | n.a. | < 14.2 | [123] |
| **CCE**<br>**COC** | Sweat | Not applicable | • Digestion<br>• pH adjustment<br>Centrifugation | DI-SPME | • Coating: PDMS<br>• Extraction: pH 8.5, stirring, 20 min<br>• TD: 20 min, 250 ºC | GC-MS | 5 ng/mL (12.5 ng/patch) | n.a. | < 8.6 | [124] |

| | | | | | | | | | | |
|---|---|---|---|---|---|---|---|---|---|---|
| **BE**<br>**CE**<br>**COC** | Hair | 50 mg | • Washing<br>• Digestion<br>• Evaporation<br>• Derivatization<br>• pH adjustment<br>• Dilution | DI-SPME | • Coating: PDMS<br>• Extraction: pH 9-10, stirring, 20 min<br>• TD: 20 min, 250 ºC | GC-MS | 0.1-0.5 ng/mg | 88.1-108.1 | < 14.2 | [125] |
| **AEME**<br>**CCE**<br>**COC**<br>**ECGME** | Saliva | 2 mL | • pH adjustment<br>• Dilution | HF-LPME | • Extraction: 10 min (2000 rpm, pH 10.5)<br>• SLM: $CHCl_3$<br>• AP: $CHCl_3$ (10 µL) | GC-MS | 6-28 | n.a. | < 11.0 | [126] |
| **AEME**<br>**CCE**<br>**COC**<br>**ECGME** | Urine | 8 mL | • pH adjustment<br>• Dilution | HF-LPME | • Extraction: 3 min (1600 rpm, pH 10.5)<br>• SLM: $CHCl_3$<br>• AP: $CHCl_3$ (10 µL) | GC-MS | 11-48 | n.a. | < 9.5 | [127] |
| **COC** | Urine | 0.25 mL | Filtration | Online-CME | • Sorbent: C18 or C18-Phe<br>• Extraction: sampling flow rate of 0.02 mL/min, loading time of 12.5 min<br>• LD: MeOH (0.25 mL, 2.5 min) | MS | 3.9 | n.a. | < 9.1 | [84] |
| **BE**<br>**CCE**<br>**COC** | Oral fluid | 0.06 mL | • Dilution<br>• Centrifugation<br>• pH adjustment | MEPS | • Sorbent: C8/SCX<br>• Conditioning: 100 µL MeOH + 100 µL $H_2O$<br>• Samples aspirated and discarded × 6<br>• Washing: 50 µL of $H_2O$/MeOH 90:10 (v:v)<br>• Drying (0.5 min)<br>• Elution: 90 µL of DCM/IPA/$NH_4OH$ (78:20:2, v:v:v)<br>• Evaporation and redissolution<br>• Cleaning: 100 µL of eluent, 100 µL of MeOH, 100 µL of $H_2O$ × 4, 100 µL of a 0.1% COOH × 4 + 100 µL of MeOH × 4 | UPLC-MS/MS | 0.25-0.5 | n.a. | < 6.9 | [128] |
| **BE**<br>**COC** | Oral fluid | 90 µL | • Centrifugation<br>• pH adjustment<br>• Dilution | µSPE | • Sorbent: C18<br>• Washing: 100 µL $H_2O$<br>• Elution: 100 µL with MeOH containing 10 mM of COOH | LC-MS/MS | 0.3-0.5 | 79-84 | ≤ 8 | [129] |
| **BE**<br>**COC** | Urine<br><br>Plasma | Urine: 90 µL<br><br>Plasma: 180 µL | • Urine samples were diluted<br>• Sonication<br>• Centrifugation<br>• pH adjustment<br>• Dilution | µSPE | • Sorbent: C18<br>• Washing: 100 µL $H_2O$<br>• Elution: 100 µL with MeOH containing 10 mM of COOH | LC-MS/MS | 0.02-0.5 | 73-101 | ≤ 8 | [130] |
| **COC** | Oral fluid | 0.5 mL | • Dilution<br>• Salt addition<br>• Protein precipitation | US-DLLME | • DS: 1.4 mL of MeOH<br>• ES: 200 µL $CHCl_3$<br>• Extraction: 2.5% NaCl, pH 8.0 | UPLC-MS/MS | 0.25 | 101.0-102.6 | ≤ 12.9 | [99] |

| | | | | | | | | | | |
|---|---|---|---|---|---|---|---|---|---|---|
| | | | | | • Ultrasound for 5 min<br>• Centrifugation (3500 rpm, 5 min)<br>• Infranatant collected, evaporated and redisolved<br>• | | | | | |
| **COC** | Oral fluid | 0.2 mL | • Dilution<br>• pH adjustment | DPX | • Sorbent: monolithic polymer<br>• Conditioning: 200 µL 1% acetic acid in MeOH and 200 µL $H_2O$<br>• Samples aspirated and dispensed × 10<br>• Washing: 200 µL 10% IPA in $H_2O$ and dried twice with 200 µL air<br>• Elution: 100 µL 1% acetic acid in MeOH × 5 | UHPLC-MS/MS | 0.1 | 85 | ≤ 8.2 | [131] |
| **EME**<br>**BE**<br>**NC**<br>**COC** | Hair | 10 mg | • Washing<br>• Digestion<br>• PLE extraction<br>• Evaporation<br>• Redissolution<br>• Centrifugation<br>• Dilution<br>• pH adjustment<br>• Salt addition | PLE-DLLME | • DS: 500 µL of IPA<br>• ES: 200 µL of $CHCl_3$<br>• Extraction: 24% NaCl, pH 11.0, 10% IPA<br>• Ultrasound for 10 min<br>• Centrifugation (9000 rpm, 5 min, 3ºC)<br>• Infranatant collected, evaporated and redisolved | LC-HRMS/MS | 0.5-5 pg/mg | 15-47 | ≤ 19 | [100] |
| **COC**<br>**BE** | Urine | 1 mL | pH adjustment | MSPE | • Sorbent: Graphene oxide – $Fe_3O_4$<br>• Extraction: vortex (15 min, pH 6)<br>• LD: 3 mL of MeOH with 10 % ammonia, 10 min vortex | UHPLC-MS/MS | 0.02-0.2 | 80.4-86.4 | ≤ 13.7 | [104] |
| **COC** | Saliva | 0.5 mL | • pH adjustment<br>• Dilution with ACN | HP-LPME | • Extraction: 35 min (300 V, pH 6.7)<br>• SLM: cellulose cone tip<br>• OP: 1-octanol (750 µL)<br>• AP: 0.4 mol/L acetic acid<br>• LD: 250 µL of a mixture of ACN/MeOH/acetic acid (45:45:10, v/v/v) in a vortex mixer for 30 s | UHPLC-MS/MS | 0.3 | 80-104 | < 16 | [132] |
| **BE**<br>**CCE**<br>**COC**<br>**EME**<br>**NC** | Plasma | 0.05 mL | • Dilution<br>• pH adjustment | DI-SPME | • Coating: C18<br>• Extraction: pH 8, 500 rpm, 30 min, 22 ºC<br>• LD: MeOH with 0.01 % COOH, 30 min, 500 rpm, 22 ºC | UPLC-MS/MS | 0.5 | 13.2-70.2 | < 10.8 | [133] |

**Table S3 - ATS**

| **Drugs** | Matrices | Sample amount | Sample pre-treatment | Microextraction technique | Optimized experimental conditions | Instrumental system | LOD (µg/L) | Absolute Recovery (%) | Precision (%) | Ref |
|---|---|---|---|---|---|---|---|---|---|---|
| **AMP**<br>**4-MAMP** | Urine | 1 mL | • Filtration<br>• Dilution<br>• pH adjustment<br>• Derivatization | EE-SDME | • Solvent: DCM (2 µL)<br>• Extraction: 4 min (-4 V, pH 7) | GC-FID | 0.14-0.27 | 82.7-96.2 | ≤ 12.8 | [136] |
| | | | | HS-SPME | • Coating: PDMS-DVB<br>• Extraction: pH 7, 40 min (600 rpm, 60 ºC)<br>• TD: 5 min, 250 ºC | | 0.05-0.09 | 90.7-112.5 | ≤ 8.5 | |
| **MET** | Urine | 5 mL | • pH adjustment<br>• Centrifugation | dSPE | • Sorbent: ZIFs (40 mg)<br>• Extraction: 5 min (2000 rpm)<br>• Centrifugation<br>• Desorption: 400 µL MeOH (sonication for 10 min)<br>• Centrifugation | HPLC-UV | 10 | 99.83 | ≤ 5.1 | [137] |
| **AMP**<br>**MET** | Plasma | 6 mL | • Centrifugation<br>• pH adjustment<br>• Protein precipitation<br>• Dilution | AA-EME | • Solvent: $CHCl_3$ and Ph-EtOH (250 µL)<br>• 8 air-agitation cycles (pH 12)<br>• Centrifugation (5000 rpm, 2 min) | HPLC-UV | 2.0-5.0 | 63-66 | ≤ 8.4 | [140] |
| **AMP**<br>**MET**<br>**MDA**<br>**MDMA**<br>**MDEA**<br>**PTM** | Urine | 5 mL | • Filtration<br>• Dilution<br>• pH adjustment | HS-SPME | • Coating: MWCNTs-COOH<br>• Extraction: pH 10, 20 min, 10 % NaCl (m/v) (900 rpm, 80 ºC)<br>• TD: 5 min, 250 ºC | GC-MS | 0.2-1.3 | 88-107 | ≤ 9.8 | [141] |
| **MET** | Urine | 4 mL | • Derivatization<br>• Dilution | SHS-HLLME | • Solvent: DPA (100 µL)<br>• Addition of 100 µL of a 6 mol $L^{-1}$ HCl solution<br>• Extraction: shaking for 10 s and water bath for 2 min (40 ºC)<br>• Addition of 100 µL of a 10 mol $L^{-1}$ NaOH solution<br>• Centrifugation (2 min) | GC-MS | 1.5 | 97.4-101.2 | ≤ 7.8 | [142] |
| **4-MAMP**<br>**AMP**<br>**FMC**<br>**MDA**<br>**MDEA**<br>**MDMA**<br>**MET** | Urine | 2 mL | • Dilution<br>• pH adjustment<br>• Salt addition<br>• Derivatization | DLLME | • DS: 350 µL of a 1:2.5 (v:v) $CHCl_3$/MeOH (Rapid injection)<br>• Centrifugation (4400 rpm, 4 min)<br>• Infranatant injected | GC-MS | 2-50 | 92-115 | ≤ 14.8 | [143] |

| | | | | | | | | | |
|---|---|---|---|---|---|---|---|---|---|
| | Blood | | • Protein precipitation<br>• Centrifugation<br>• Dilution<br>• pH adjustment<br>• Salt addition<br>• Derivatization | | | | | | |
| **AMP** | Urine | 0.2 mL | • pH adjustment<br>• Dilution<br>• Salt addition | µSPE | • Sorbent: MIP<br>• Conditioning: $H_2O$ (100 µL)<br>• Extraction: 10 min (2500 rpm)<br>• Washing: $H_2O$ (200 µL)<br>• Elution: 200 µL (MeOH, 6 min) | LC-MS/MS | 1 | 80 | ≤ 8.5 | [144] |
| **AMP**<br>**MDA**<br>**MDMA**<br>**MET** | Whole Blood | 0.2 mL | • pH adjustment<br>• Dilution<br>• Salt addition | DLLME | • DS: 1.3 mL MeOH<br>• Vortex (2 min)<br>• Centrifugation (4000 rpm, 5 min)<br>• Supernatant collected<br>• ES: 0.2 mL DCM<br>• pH adjustment<br>• NaCl addition<br>• Sonication for 2 min<br>• Centrifugation (3500 rpm, 5 min)<br>• Infranatant injected | GC-MS | 10 | 77.0-92.4 | ≤ 12.8 | [145] |
| **MPH** | Urine | 2.5 mL | • Dilution<br>• Centrifugation<br>• pH adjustment<br>• Salt addition | SBME | • Extraction: 25 min (650 rpm, pH 11.6, 25ºC, 30 % NaCl (w/v))<br>• SLM: 1-octanol<br>• AP: pH 4.0, 30 µL | HPLC-UV | 15 | n.a. | ≤ 3.9 | [138] |
| **MDMA**<br>**MET** | Urine | 2 mL | • Salt addition<br>• pH adjustment | HS-SPME | • Coating: MWCNTs/ILs<br>• Extraction: pH 11, 20 min, 20 % NaCl (w/v) (500 rpm, 80 ºC)<br>• TD: 4 min, 250 ºC | GC-FID | 0.097-0.39 | n.a. | ≤ 7.0 | [139] |
| **AMP**<br>**MBDB**<br>**MDA**<br>**MDEA**<br>**MDMA**<br>**MET** | Sweat | Cotton pad (0.5 × 0.5 cm) 4-6 times rubbing on the forehead | • Acidic extraction<br>• pH adjustment | HS-SPME | • Coating: PDMS<br>• Extraction: 10 min (90ºC) + 3 min (90ºC) for derivatization<br>• TD: 3 min, 250 ºC | GC-MS | 0.07-0.27 ng/pad | 95.1-100.9 | ≤ 9.9 | [89] |
| **AMP**<br>**MET** | Urine | 1 mL | • pH adjustment<br>• Dilution<br>• Salt addition | HF-LPME | • SLM: DHE<br>• AP: 30 µL HCl 0.01 M | GC-MS | 10-20 | 50.9-76.0 | ≤ 19.2 | [146] |

| | | | | | | | | | | |
|---|---|---|---|---|---|---|---|---|---|---|
| | | | | | • Extraction: 60 min (1200 rpm, pH > 12, 10 % NaCl (w/v), 20-24ºC)<br>• Derivatization<br>• Evaporation<br>• Redissolution | | | | | |
| **MET** | Urine | 10 mL | Filtration | DLLME | • DS: 0.50 mL MeOH<br>• ES: ILs (50 μL)<br>• Centrifugation (7000 rpm, 16 min)<br>• Infranatant injected | HPLC-UV | 1.7 | 80.5-81.6 | ≤ 6.4 | [147] |
| **MET** | Urine | 4 mL | • pH adjustment<br>• Dilution<br>• Filtration | HF-EME | • Extraction: 20 min (1000 rpm, pH 7, 60 V)<br>• SLM: 1-octanol containing GO<br>• AP: 20 μL 1-octanol | GC-FID | 2.4 | n.a. | ≤ 7.2 | [148] |
| | Hair | 2 g | • Washing<br>• Drying<br>• Alkaline digestion<br>• pH adjustment<br>• Dilution<br>• Filtration | | | | | | | |
| **AMP**<br>**EPE**<br>**MET** | Oral fluid | 0.125 mL | • pH adjustment<br>• Dilution<br>Derivatization | In tube-SPME | • Coating: 35 % BP, 65 % PDMSE funcionalized with MWCNTs<br>• Extraction: 20 μL (1 min)<br>• Washing (20 μL)<br>• LD: ACN:$H_2O$ 70:30 (v/v) | HPLC-DAD | 0.5-0.8 | n.a. | ≤ 5.0 | [149] |
| **FEN**<br>**DIE**<br>**SIB** | Urine | 5 mL | • Filtration<br>• pH adjustment | DLLME | • DS: 1 mL of MeOH<br>• ES: 300 μL $CHCl_3$<br>• Manual agitation (15 s)<br>• Centrifugation (3000 rpm,5 min)<br>• Infranatant injected | GC-MS | 0.05-0.1 | 82.1-104.2 | ≤ 15.3 | [150] |
| **AMP**<br>**MET** | Urine | 5 mL | Not necessary | CCSHLLE-DLLME-SFO | • Pour NaCl into a syringe barrel<br>• Mix sample with 1 mL of ACN<br>• Pass the mixture into the syringe barrel at a flow of 0.6 mL/min<br>• Salting out occurs<br>• Supernatant collected and transferred to a test tube<br>• ES: 34 μL 1-undecanol | HPLC–UV | 0.5-2 | 78-84 | ≤ 5 | [151] |

| | | | | | | | | | | |
|---|---|---|---|---|---|---|---|---|---|---|
| | | | | | • DS: $K_2CO_3$ solution (2% w/v, 5.00 mL)<br>• Rapid injection<br>• Centrifugation (4200 g, 4 min)<br>• Ice bath (5 min)<br>• SFO collected and injected | | | | | |
| **MET** | Urine | 1 mL | • pH adjustment<br>• Dilution<br>• Salt addition | HF-SLPME | • SLM: 1-octanol<br>• AP: MOF/GO dissolved in 1-octanol<br>• Extraction: 30 min (900 rpm, pH 11.0, 30 % NaCl w/v)<br>• LD: 40 μL MeOH (vortex for 3 min) | TC-ESI-MS | 0.5 | 98.9-103.8 | ≤ 9.52 | [152] |
| **AMP**<br>**MET**<br>**MPTM**<br>**OMMA**<br>**PEA** | Urine | Information not available | Not necessary | SDME | • Solvent: DCM (2 μL)<br>• DP: 0.1 M NaOH<br>• SLM: 1-octanol<br>• AP: 20-mM AmFo buffer (pH 2.5)<br>• Extraction: 10 min (-4 V, pH 7) | CE-MS | 2-5 nM | n.a. | ≤ 13 | [153] |
| **AMP**<br>**MDA**<br>**MDMA**<br>**MET** | Blood | 0.5 mL | • Protein precipitation<br>• Centrifugation<br>• Dilution<br>• Salt addition<br>• pH adjustment | DLLME | • DS: 0.25 mL of MeOH<br>• ES: 100 μL $CHCl_3$<br>• Rapid injection<br>• Sonication (1 min)<br>• Centrifugation (4000 rpm,5 min)<br>• Infranatant collected, evaporated and redissolved | UPLC-MS/MS | 0.5-2 | 3-124 | ≤ 15 | [90] |
| **AMP**<br>**MET** | Urine | 5 mL | • Filtration<br>• Dilution | EE-DI-SPME | • Coating: OMC/Nafion<br>• Extraction: 20 min (750 rpm, -0.9 V)<br>• TD: 4 min, 260 ºC | GC-FTD | 1.2-4.8 | 66.2-79.5 | ≤ 8.5 | [154] |
| | Serum | 3 mL | Dilution | | | | | | | |
| **AMP**<br>**MET** | Urine | 5 mL | • Dilution<br>• pH adjustment | EM-DI-SPME | • Coating: NCF<br>• SLM: 85% NPOE and 15% TEHP<br>• AP: $H_2O$, pH 7<br>• Extraction: 15 min (700 rpm, pH 4), 150 V<br>• TD: 2 min, 280 ºC | GC-FID | 1.0-2.0 | 9.0-18.8 | ≤12.5 | [155] |
| | Whole blood | 1 mL | | | | | | | | |

| | | | | | | | | | | |
|---|---|---|---|---|---|---|---|---|---|---|
| **AMP**<br>**MDEA**<br>**MDMA**<br>**MET** | Oral fluid | 0.12 mL | • Dilution<br>• pH adjustment<br>• Protein precipitation<br>• Sonication<br>• Centrifugation | MEPS | • Sorbent: C18<br>• Conditioning: 250 μL MeOH (× 2) + 250 μL $H_2O$/ MeOH (80:20, v/v, × 2)<br>• Samples aspirated and discarded × 5<br>• Washing: 100 μL 50 mM $NH_3$ in $H_2O$/MeOH (90:10, v/v; × 3)<br>• Elution: 100 μL 5 mM COOH in MeOH (× 5) | LC-MS/MS | 0.3-1 | 18-90 | ≤ 18 | [91] |
| **AM**<br>**MACT**<br>**MDA**<br>**MDMA**<br>**MET** | Urine Blood | 1 mL | • Dilution<br>• pH adjustment | HF-LPME | • SLM: toluene<br>• AP: toluene (10 μL)<br>• Extraction: 10 min (500 rpm, pH 13.0, 30 ºC) | GC-MS | 1-5 | 79.3-94.9 | ≤ 4.5 | [92] |
| | | | | DLLME | • ES and DS: 100 μL toluene<br>• Sonication (3 min) and manual shaking<br>• Centrifugation (10000 rpm,3 min)<br>• Supernatant collected<br>• For blood samples, 10 mg of NaCl was added to break emulsion | | 1-4 | 79.9-100.3 | ≤ 5.7 | |
| **MET** | Urine | 3 mL | pH adjustment | DLLME | • DS: 200 μL ACN<br>• ES: 70 μL ILs<br>• Extraction: NaCl 20 % (w/v) pH 8.0, vortex 1 min + 6 min (50 ºC) + ice bath 11 min<br>• Centrifugation (8000 rpm,4 min)<br>• Infranatand collected and redissolved in MeOH | HPLC-UV | 10 | 88.7 | ≤ 4.7 | [156] |
| **MET** | Urine | 1 mL | • Dilution<br>• Shaking<br>• Centrifugation<br>• Protein precipitation<br>• pH adjustment | MDSPE | • Coating: magnetic MWCNTs<br>• Extraction: 10 min (3000 rpm, 10 % NaCl, pH 13)<br>• LD: 10 min sonication, 100 μL ethyl acetate | GC-MS | 0.044 | 79.5-86.6 | ≤ 7.4 | [157] |
| | Blood | 1 mL | • Protein precipitation<br>• Shaking<br>• Centrifugation | | | | | | | |
| **EPE**<br>**MET** | Urine | 5 mL | • Filtration<br>• Dilution<br>• pH adjustment<br>• Salt addition | HS-SPME | • Coating: MWCNTs/ILs/Nafion | GC-FID | 0.33-0.6 | 91.5-109.0 | ≤ 6.1 | [158] |

| | | | | | | | | | |
|---|---|---|---|---|---|---|---|---|---|
| | | | | | • Extraction: 40 min (1000 rpm, 10-30 % NaCl, 2-10 mol/L NaOH, 60-85 ºC)<br>• TD: 275 ºC | | | | | |
| **AMP**<br>**MET** | Urine | 0.1 mL | Not necessary | MEPS | • Sorbent: C8<br>• Activation: 100 µL MeOH (× 3)<br>• Conditioning: 100 µL $H_2O$ (× 3)<br>• Samples aspirated and discarded x 5<br>• Washing: 100 µL $H_2O$ (× 2) and 100 µL of 5 % MeOH (× 1)<br>• Elution: 50 µL 0.1% COOH in MeOH and injection in MS system. | MS | 1.5-6.0 | 91.7-106.7 | ≤ 17.1 | [40] |
| **AMP**<br>**DIE**<br>**FEN** | Plasma | 2 mL | • Protein precipitation<br>• Vortex and centrifugation<br>• pH adjustment<br>• Derivatization | DI-SPME | • Coating: PDMS<br>• Extraction: 35 min (1200 rpm, 10-30 % NaCl pH 10.2, 20ºC)<br>• TD: 15 min, 260ºC | GC-MS | 1.0-2.0 | 46.4-84.5 | ≤ 15.0 | [159] |
| **AMP**<br>**MET** | Hair | 20 mg | • Washing<br>• Drying<br>• Digestion<br>• Salt addition | HF-SPME | • Coating: PDMS/DVB<br>• Extraction: 10 min (90ºC) TD: 5 min, 250ºC | GC-MS | 0.3-2.0 ng/mg | n.a. | ≤ 13.1 | [160] |
| **AMP**<br>**MET** | Urine | 2 mL | • Centrifugation<br>• Filtration<br>• Dilution<br>• pH adjustment<br>• Salt addition | DLLME-SFO | • DS: 300 µL ACN<br>• ES: 30 µL 1-undecanol<br>• Extraction: $K_2CO_3$ 2 % (w/v) pH 10.2<br>• Rapid injection<br>• Centrifugation (5000 rpm,4 min)<br>• Ice bath (5 min)<br>• SFO collected and injected | HPLC-UV | 2-8 | 58.5-62.4 | ≤ 7.8 | [161] |
| **MET** | Urine | 5 mL | • Dilution<br>• pH adjustment | EE-DI-SPME | • Coating: CAR/PDMS<br>• Extraction: 20 min (1000 rpm, pH 7, 12 V)<br>• TD: 3 min, 250ºC | GC-MS | 0.25 | 89.9 | ≤ 6.12 | [162] |
| **AMP**<br>**MDA**<br>**MDEA**<br>**MDMA**<br>**MET** | Urine | 1 mL | • Filtration<br>• Derivatization<br>• Dilution<br>• Salt addition | DI-SPME | • Coating: PDMS/DVB<br>• Extraction: 40 min (600 rpm, 60ºC, pH 7)<br>• TD: 3 min, 250ºC | GC-MS | 0.01-0.09 | n.a. | ≤ 10.2 | [163] |

| | | | | | | | | | | |
|---|---|---|---|---|---|---|---|---|---|---|
| **AMP<br>EPE<br>MBDB<br>MDA<br>MDEA<br>MDMA<br>MET<br>PEPE** | Urine | 4 mL | • Centrifugation<br>• Filtration<br>• pH adjustment<br>• Dilution | DLLME | • DS: 1400 μL IPA<br>• ES: 600 μL DCM<br>• Extraction: pH ≥ 11.5<br>• Rapid injection<br>• Centrifugation (10000 rpm,5 min)<br>• Infranatant collected, evaporated and redissolved | CE-TOF-MS | 0.25-0.50 | 61-75 | n.a. | [93] |
| **MDMA** | Urine | 4 mL | • Dilution<br>• Salt addition<br>• pH adjustment | DLLME | • DS: 1505 μL ACN<br>• ES: 606 μL $CH_2Br_2$<br>• Extraction: 30%-$NH_3$ pH ≥ 11.5<br>• Rapid injection<br>• Centrifugation (9500 rpm,5 min)<br>• Infranatant collected, evaporated and redissolved | CE-UV | 1.0-3.47 | 91.1 | ≤ 6.7 | [164] |
| **AMP<br>MDMA<br>MET** | Urine | 12 mL | • Salt addition<br>• pH adjustment | INAT | • Coating: MIP<br>• Washing: 2 mL $H_2O$<br>• Extraction: 30 min (4 % NaCl (w/v), pH 8)<br>• TD: 3 min, 280ºC | GC-FID | 12-42 | 72-92 | ≤ 8.7 | [165] |
| **MDA<br>MDEA<br>MDMA<br>MDPA** | Urine | 4 mL | • pH adjustment<br>• Dilution | USAEME | • ES: 14 μL toluene<br>• Extraction: pH 10, sonication (30 s), 25±3ºC<br>• Centrifugation (3500 rpm, 5 min)<br>• Supernatant collected and injected | GC-FID | 0.2-0.4 | 38.0-71.7 | ≤ 13.1 | [166] |
| **AMP<br>FEN<br>MDA<br>MDMA<br>MET** | Hair | 50 mg | • Washing<br>• Drying<br>• Digestion<br>• Salt addition | HF-LPME | • SLM: DHE<br>• AP: 15 μL of HCl 0.1 mol/L<br>• Extraction: 45 min (1000 rpm, 10 % NaCl)<br>• Evaporation<br>• Derivatization<br>• Evaporation<br>• Redissolution | GC-MS | 0.01-0.04 ng/mg | 36.0-89.6 | ≤ 11.4 | [167] |
| **MDA<br>MDEA<br>MDMA<br>MDPA** | Urine | 1 mL | • Dilution<br>• pH adjustment | DLLME | • DS: 1000 μL acetone<br>• ES: 30 μL $CS_2$<br>• Extraction: pH 10<br>• Rapid injection<br>• Centrifugation (3000 rpm,3 min)<br>• Infranatant collected | GC-FID | 0.3-0.8 | 27.1-55.1 | ≤ 11.5 | [168] |

| | | | | | | | | | |
|---|---|---|---|---|---|---|---|---|---|
| **MET MDMA** | Urine | 10 mL | • Centrifugation<br>• pH adjustment<br>• Salt addition | SPE-DLLME | SPE<br>• Sorbent: MIP<br>• Conditioning: 2 mL ACN + 5 mL $H_2O$<br>• Sample: 3% (w/v) NaCl with pH 8<br>• Washing: 1 mL $H_2O$<br>• Elution: 1 mL MeOH<br>• Derivatization<br><br>DLLME<br>• ES: 30 µL $C_5H_9ClO_2$<br>• Rapid injection into 5 mL aqueous solution<br>• Centrifugation (5000 rpm, 6 min)<br>• Infranatant collected | GC-MS | 2-18 | 78-89 | ≤ 7.7 | [169] |
| **AMP 4-MAMP** | Urine | 1 mL | • pH adjustment<br>• Salt addition<br>• Dilution | DLLME-SFO | • DS: 56.5 µL SDS<br>• ES: 31 µL 1-undecanol<br>• Extraction: pH 6.4, 0.3% (w/v) NaCl, 0.08 M surfactant<br>• Rapid injection and vortex (30 s)<br>• Centrifugation (4500 rpm,5 min)<br>• Ice bath (5 min)<br>• SFO collected and injected | HPLC-UV | 2-3 | 24-28 | ≤ 5.6 | [170] |
| **4-MAMP MDA MDMA DOI)** | Whole blood and post mortem blood | 80 µL | Not necessary | EME | • SLM: ENB<br>• AP: 10 µl 10 mM acetic acid<br>• Extraction: 5 min (15 V<br>• Dilution | UPLC-MS/MS | 0.125-2.609 | 10-30 | ≤ 26 | [171] |
| **EPE** | Horse urine | 10 mL | pH adjusment | SDME | • Solvent: 5 µL $CHCl_3$/MeOH (90:10)<br>• Extraction: 20 min (25 ºC, pH 11, 500 rpm)<br>• Evaporation and redissolution | OT-CEC | 7.71-8.27 | n.a. | ≤ 2 | [94] |
| **AMP, 4-MAMP, MDA MDMA** | Urine | 2 mL | • Filtration<br>• pH adjustment<br>• Dilution | MDSPE | • Sorbent: Fe3O4/SiO2/poly(MAA-co-EDMA<br>• Extraction: vortex (5 s, pH 9.2)<br>• Washing: 0.5 mL H2O (× 5) | CE-UV | 43-105 | 85.4-109.7 | ≤ 10.3 | [95] |

| | | | | | | | | | |
|---|---|---|---|---|---|---|---|---|---|
| | | | | | • LD: 0.04 mL of acetone containing 0.5% COOH (v/v) with 10 s vortex. | | | | |
| **AMP, 4-MAMP, MDA, MDMA, MBDB** | Urine | 3 mL | pH adjustment | EME | • SLM: NPOE containing 15% TEHP<br>• AP: 15 µl 100 mM HCl<br>• Extraction: 7 min (1000 rpm, 250 V, 10 mM HCl) | HPLC-DAD | 5-10 | 54-70 | ≤ 10.2 | [172] |
| **AMP<br>4-MAMP** | Urine | 10 mL | No sample pre-treatment needed | EE-SPME | • Coating: MWCNTs/Nafion<br>• Extraction: 10 min (750 rpm, 0.6 V)<br>• TD: 4 min, 260ºC | GC-FID | 0.12-0.26 | 1.3-4.0 | ≤ 8.0 | [173] |
| **AMP<br>4-MAMP<br>MDA<br>MDMA** | Urine | 3 mL | • pH adjustment<br>• Salt addition | HF-LPME | • SLM: o-xylene<br>• AP: o-xylene<br>• Extraction: 20 min (1000 rpm, pH 12.5, 30 % NaCl, 30ºC) | GC-FID | 8-82 | n.a. | ≤14.1 | [174] |
| **AMP<br>4-MAMP<br>MDA<br>MDMA<br>MDE<br>MBDB<br>MDPA** | Hair | 10 mg | • Washing<br>• Drying<br>• Digestion<br>• Salt addition | HS-SPME | • Coating: PDMS<br>• Extraction: 10 min (90ºC)<br>• Derivatization<br>• TD: 4 min, 260ºC | GC-MS | 0.06-0.12 ng/mg | 3.1-16.6 | ≤ 9.9 | [96] |
| **MDMA** | Hair | 2 g | • Washing<br>• Drying<br>• Digestion<br>• Filtration<br>• pH adjustment | SDME | • Solvent: 350 µL1-octanol<br>• Extraction: 3 min (0.1 mL Titron X-100, pH 11, 1000 rpm)<br>• LD: 10 µL $H_2O$ pH 12 (20 min, 600 rpm) | HPLC-DAD | 0.1 | n.a. | ≤ 5.4 | [175] |
| **AMP<br>4-MAMP** | Urine | 2 mL | • Salt addition<br>• pH adjustment<br>• dilution<br>• Filtration | HS-SPME | • Coating: ILs<br>• Extraction: 20 min (35 % NaCl, NaOH 4 M, 1200 rpm, 50ºC)<br>• Derivatization<br>• TD: 220ºC | GC-MS | 0.1-0.5 | n.a. | ≤ 11.3 | [176] |
| **AMP<br>4-MAMP<br>MDA<br>MDMA** | Whole blood | 1 mL | • Dilution<br>• Filtration<br>• pH adjustment<br>• Salt addition | HS-SPME | • Coating: PA<br>• Derivatization<br>• Extraction: 15 min (10 % NaCl, $K_2CO_3$ 5 M, 250 rpm, 80ºC)<br>• Derivatization<br>• TD: 6 min, 275ºC | GC-MS | 10 | n.a. | ≤ 30.6 | [177] |
| **AMP** | Urine | 0.5 mL | Dilution | DPSC | • Sorbent: C18/monolithic silica | HPLC-DAD | 100 | ~ 100 | ≤ 10.4 | [178] |

| | | | | | | | | | | |
|---|---|---|---|---|---|---|---|---|---|---|
| **4-MAMP**<br>**MDMA**<br>**MDA** | | | | | • Conditioning: 0.5 mL ACN + 0.5 mL $H_2O$<br>• Centrifugation (3000 rpm, 5 min)<br>• Extraction: sample + 0.4 mL buffer (pH 12)<br>• Centrifugation (3000 rpm, 5 min)<br>• Washing: 0.5 mL ACN and an alkaline buffer (pH 12; 10:90, v/v)<br>• Elution: 0.2 mL ACN–phosphate buffer (pH 3.0, containing 20 mM sodium octanesulfate) (25:75, v/v) | | | | | |
| **AMP**<br>**MDA** | Urine | 4 mL | • Filtration<br>• pH adjustment<br>• Salt addition | HS-HF-LPME | • AP: *n*-nonanol containing derivatizing reagent<br>• Extraction: 30 min (750 rpm, KOH 4 M, 36 % NaCl, 95 ºC) | GC-MS | 0.25-1.00 (LOQ) | 5.2-19.6 | ≤ 4 | [179] |
| **4-MAMP**<br>**MDMA** | Serum | 0.2 mL | • Dilution<br>• pH adjustment | HS-SPME | • Coating: PPy-DS<br>• Extraction: 10 min (60 ºC) + 60 min (4 M NaCl, 600 rpm, pH 12, 55 ºC)<br>• TD: 70 s, 240 ºC | IMS | 5-8 | n.a. | ≤ 7.2 | [180] |
| **4-MAMP**<br>**MDMA** | Urine | 1 mL | Dilution | HS-SPME | • Coating: PDMS<br>• Derivatization<br>• Extraction: 20 min (4 M NaCl, 600 rpm, pH 12, 90ºC)<br>• TD: 1 min, 225ºC | GC-MS | 50-100 | n.a. | ≤ 14.4 | [180] |
| **AMP**<br>**MET** | Urine | 2.5 mL | • Filtration<br>• pH adjustment<br>• Salt addition<br>• Dilution | HS-SDME | • Solvent: $H_3PO_4$ 0.05 M<br>• Extraction: 15 min (80ºC) + 20 min (4 M NaOH, 10 % NaCl, 1200 rpm) | HPLC-UV | 0.3 | n.a. | ≤ 12.5 | [181] |
| **AMP**<br>**MET** | Urine | 3 mL | • Dilution<br>• pH adjustment<br>• Filtration | SDME | • Extraction: 20 min (0.4 mL *n*-C6, 25 ºC, 0.5 M NaOH, 1200 rpm)<br>• Back-extraction: 5 µL of 0.02 M $H_3PO_4$ (20 min, 1200 rpm) | HPLC-UV | 0.5 | n.a. | ≤ 5.4 | [182] |
| **AMP**<br>**MET** | Hair | 0.08 mg | • Washing<br>• Drying<br>• Digestion<br>• pH adjustment | HS-SPME | • Coating: PDMS<br>• Derivatization<br>• Extraction: 20 min (1 M NaOH, 70ºC)<br>• TD: 3 min, 250ºC | GC-MS | 0.25-0.625 ng/mg | 2.8-17.5 | ≤ 26.4 | [183] |

| | | | | | | | | | |
|---|---|---|---|---|---|---|---|---|---|
| **EPE**<br>**4-MAMP** | Urine | 5 mL | • Dilution<br>• pH adjustment<br>• Salt addition | HS-SPME | • Coating: DM-β-CD/OH-TSO<br>• Extraction: 35 min (pH 10-14, 50-80ºC, 40 % NaCl, 1000 rpm)<br>• TD: 250ºC | GC-FID | 0.33-0.60 | n.a. | ≤ 5.0 | [184] |
| **AMP**<br>**4-MAMP**<br>**MDA**<br>**MDMA**<br>**MDEA** | Urine | 2 mL | • pH adjustment<br>• Salt addition | HS-SPME | • Coating: PDMS<br>• Extraction: 30 min (pH 10-14, 95 ºC, 36 % NaCl, 600 rpm)<br>• Derivatization<br>• TD: 7 min, 260ºC | GC-MS | 0.016-0.193 | 16.9-19.6 | ≤ 6.5 | [185] |
| **AMP**<br>**MET** | Serum | 3 mL | • pH adjustment<br>• Dilution | HS-SPME | • Coating: PDMS/DVB<br>• Extraction: 30 min (pH 10, 1000 rpm)<br>• Derivatization<br>• LD: 80 % ACN, 15 min | LC-MS/MS | 0.04-0.3 | 3-8 | ≤ 18 | [186] |
| **AMP**<br>**PHD**<br>**PMT**<br>**PDM**<br>**DEP**<br>**FCF**<br>**NPEPE**<br>**EPE**<br>**PTM**<br>**MET**<br>**4-EAMP**<br>**FFA**<br>**MDA**<br>**MDMA**<br>**MDEA**<br>**MBDB,**<br>**meperidi**<br>**ne**<br>**BZP**<br>**DAMP**<br>**MTA**<br>**OMMA**<br>**MEPE** | Urine | 0.5 mL | • Dilution<br>• pH adjustment<br>• Salt addition | DI-SPME | • Coating: PDMS/DVB<br>• Extraction: 30 min (pH 12, 40ºC)<br>• TD: 260ºC, 2 min | GC-MS | 50-1000 | n.a. | ≤ 10.9 | [97] |
| **EPE** | Urine | 1 mL | • Dilution<br>• pH adjustment | DI-SPME | • Coating: DVB/CAR/PDMS<br>• Extraction: 30 min (pH 10, 80ºC)<br>• TD: 260ºC | IMS | 50 | 101 | ≤ 5 | [187] |
| **AMP**<br>**MET** | Urine | 0.5 mL | Dilution | DI-SPME | • Coating: Carbowax<br>• Extraction: 15 min | HPLC-FLD | 100-1000 | n.a. | ≤ 21 | [188] |

| | | | | | | | | | | |
|---|---|---|---|---|---|---|---|---|---|---|
| **MDMA** | | | | | • Derivatization<br>• LD: 0.2 mL ACN, 5 min | | | | | |
| **AMP**<br>**MET**<br>**MDA**<br>**MDMA**<br>**MDE**<br>**MBDB** | Hair | 20 mg | • Washing<br>• Drying<br>• Digestion<br>• pH adjustment<br>• Salt addition | HS-SPME | • Coating: PDMS<br>• Extraction: 5 min (90ºC, pH 10.3)<br>• TD: 250ºC, 3 min | GC-MS | 0.35-1.61 ng/mg | n.a. | ≤ 19.2 | [98] |
| **AMP**<br>**MET**<br>**MDA**<br>**MDMA**<br>**MDEA** | Urine | 1 mL | • pH adjustment<br>• Salt addition | HS-SPME | • Coating: PDMS<br>• Extraction: 30-45 min (60-90ºC)<br>• TD: 220ºC, 1 min | GC-FID | 30-40 | 5.1-47 | n.a. | [189] |
| **AMP**<br>**MET** | Saliva | 1 mL | • Centrifugation<br>• Dilution<br>• pH adjustment | HS-SPME | • Coating: PDMS<br>• Derivatization<br>• Extraction: 20 min (70ºC)<br>• TD: 250ºC, 15 min | GC-MS | 0.5-5.0 | 68.2-104.4 | ≤ 7.7 | [190] |
| **AMP**<br>**MET**<br>**MDA**<br>**MDMA**<br>**MDEA** | Urine | 4 mL | pH adjusment | DI-SPME | • Coating: PDMS<br>• Extraction: 12 min (25ºC, pH 10)<br>• LD: 0.25 mM ammonium acetate in MeOH | FAIMS-MS | 0.2-7.5 | n.a. | n.a. | [191] |
| **AMP**<br>**MET**<br>**MDA**<br>**MDMA**<br>**MDEA**<br>**MBDB** | Hair | 10 mg | • Washing<br>• Drying<br>• Digestion<br>• pH adjustment | HS-SPME | • Coating: PDMS<br>• Derivatization<br>• Extraction: 5 + 10 min (50ºC, 650 rpm)<br>• Derivatization<br>• TD: 240ºC, 2 min | GC-MS | 0.01-0.17 ng/mg | 0.3-7.5 | ≤ 17.0 | [192] |
| **MET**<br>**AMP**<br>**MDA**<br>**MDMA** | Urine | 1 mL | • Derivatization<br>• pH adjustment<br>• Dilution | SBSE | • Coating: PDMS<br>• Extraction: 60 min (500 rpm, pH 3)<br>• LD: 1 mL ACN (30 min)<br>• Evaporation and redissolution | LC-MS/MS | 0.17-1.08 | 56.5-84.3 | ≤ 8.7 | [193] |
| **AMP**<br>**MET**<br>**MDA**<br>**MDMA** | Urine | 3 mL | • pH adjustment<br>• Dilution<br>• Salt addition | SBSE | • Coating: titania-OH-TSO<br>• Extraction: 20 min (700 rpm, pH 11, 20 % NaCl)<br>• LD: 60 µL 80/20 (v/v) MeOH/phosphate buffer, pH 1.5 (20 min) | HPLV-UV | 2.3-9.1 | 101.7-117.3 | ≤ 8.9 | [194] |
| **AMP**<br>**MET**<br>**MDA**<br>**MDMA** | Urine | 2 mL | • Filtration<br>• pH adjustment | SDME | • Solvent: $CHCl_3$(2 µL)<br>• DP: 0.1 M NaOH<br>• AP: (pH 10.5)<br>• Extraction: 8 min | GC-PDHID | 25-555 | n.a. | ≤ 23.5 | [195] |

| | | | | | | | | | | |
|---|---|---|---|---|---|---|---|---|---|---|
| **MDEA** | | | | | | | | | | |
| **MET**<br>**MDMA** | Urine | 1 mL | • Derivatization<br>• Dilution | HS-SPME | • Coating: PDMS<br>• Derivatization<br>• Extraction: 20 min (90ºC)<br>• TD: 225ºC, 1 min | GC-MS | 0.05-0.1 | n.a. | ≤ 14.4 | [196] |
| **AMP**<br>**MET**<br>**MDA**<br>**MDMA**<br>**MDEA** | Oral fluid | 90 μL | • Centrifugation<br>• pH adjustment<br>• Dilution | μSPE | • Sorbent: C18<br>• Extraction: 15 min (30ºC, 200 rpm)<br>• Washing: 100 μL $H_2O$<br>• Elution: 100 μL MeOH containing 10 mM COOH | LC-MS/MS | 0.05-0.2 | 63-79 | ≤ 8 | [129] |
| **AMP**<br>**MET**<br>**MDA**<br>**MDMA**<br>**MDEA** | Urine<br>Plasma | Urine: 90 μL<br>Plasma: 180 μL | • Urine samples were diluted<br>• Sonication<br>• Centrifugation<br>• pH adjustment<br>• Dilution | μSPE | • Sorbent: C18<br>• Washing: 100 μL $H_2O$<br>• Elution: 100 μL with MeOH containing 10 mM of COOH | LC-MS/MS | 0.6-2.0 | 55-103 | ≤ 10 | [130] |
| **AMP**<br>**MET**<br>**PMA**<br>**MDMA** | Urine | 1 mL | • pH adjustment<br>• Salt addition | DI-SPME | • Coating: PDMS/DVB<br>• Extraction: 60 min, 42 % NaCl (m/v), pH 12.6 (2000 rpm)<br>• LD: 65 μL MeOH + 10 μL HCl:MeOH (1:9) (2000 rpm)<br>• Evaporation and derivatization<br>• Evaporation and redissolution | GC-MS | 5-10 | 2-80 | ≤ 13 | [197] |

| | | | | | | | | | | |
|---|---|---|---|---|---|---|---|---|---|---|
| **MET** | Oral fluid | 0.2 mL | • Dilution<br>• pH adjustment | DPX | • Sorbent: monolithic polymer<br>• Conditioning: 200 µL 1% acetic acid in MeOH and 200 µL $H_2O$<br>• Samples aspirated and dispensed x 10<br>• Washing: 200 µL 10% IPA in $H_2O$ and dried twice with 200 µL air<br>• Elution: 100 µL 1% acetic acid in MeOH × 5 | UHPLC-MS/MS | 0.5 | 71 | ≤ 4.2 | [131] |
| **AMP**<br>**MET**<br>**MDMA** | Hair | 10 mg | • Washing<br>• Digestion<br>• PLE extraction<br>• Evaporation<br>• Redissolution<br>• Centrifugation<br>• Dilution<br>• pH adjustment<br>• Salt addition | PLE-DLLME | • DS: 500 µL of IPA<br>• ES: 200 µL of $CHCl_3$<br>• Extraction: 24% NaCl, pH 11.0, 10% IPA<br>• Ultrasound for 10 min<br>• Centrifugation (9000 rpm, 5 min, 3ºC)<br>• Infranatant collected, evaporated and redissolved | HPLC-HRMS/MS | 0.2-1 pg/mg | 43-56 | ≤ 22 | [100] |
| **AMP**<br>**MAMP** | Urine | 1 mL | pH adjustment | MSPE | • Sorbent: Graphene oxide – $Fe_3O_4$<br>• Extraction: vortex (15 min, pH 6)<br>• LD: 3 mL of MeOH with 10 % ammonia, 10 min vortex | UHPLC-MS/MS | 0.02-0.03 | 83.6-105.5 | ≤ 13.1 | [104] |
| **MPH** | Urine | 6 mL | • pH adjustment<br>• Centrifugation | DSPE | • Sorbent: Silica-functionalized nano-graphene oxide composite<br>• Extraction: 4000 rpm (15 min, pH 9)<br>• LD: 0.4 mL of MeOH by sonication (20 min) | HPLC-UV | 30.0 | 95.3 | ≤ 6.7 | [198] |

| | | | | | | | | | | |
|---|---|---|---|---|---|---|---|---|---|---|
| **AMP**<br>**MAMP**<br>**FEN**<br>**MDMA**<br>**MDA**<br>**MDEA** | Whole blood | 0.5 mL | • Dilution<br>• pH adjustment | HP-LPME | • SLM: dihexyl ether<br>• AP: 50 µL of 0.1 mol/L HCl (pH 3)<br>• Extraction: 30 min (1500 rpm, pH 12, 45ºC)<br>• Evaporated, derivatized and redissolved | GC-MS | 1-3 | 69-110 | ≤ 10.0 | [199] |
| **MAMP** | Urine | 6 mL | • pH adjustment<br>• Centrifugation | DSPE | • Sorbent: carbon-based conductive polypyrrole nanocomposite<br>• Extraction: 6000 rpm (10 min, pH 9)<br>• LD: 0.3 mL of MeOH by sonication (10 min) | HPLC-UV | 9 | 99.8 | ≤ 6.0 | [200] |
| **MPH** | Urine | 1.5 mL | • Dilution<br>• pH adjustment | EME | • Extraction: 20 min (pH 4, 180 V) 1000 rpm<br>• SLM: Porous Aromatic Framework-48 with the nitro functional groups with NPOE<br>• AP: 100 mmol/L HCl (20 µL) | IMS | 3.6 | 94-99 | ≤ 5.2 | [105] |

**Table S4 - Cannabinoids**

| **Drugs** | Matrices | Sample amount | Sample pre-treatment | Microextraction technique | Optimized experimental conditions | Instrumental system | LOD (µg/L) | Absolute recovery (%) | Precision (%) | Ref |
|---|---|---|---|---|---|---|---|---|---|---|
| **THC**<br>**11-OH-THC**<br>**THC-COOH** | Plasma | 0.25 mL | • Protein precipitation<br>• Dilution<br>• Centrifugation<br>• Evaporation<br>• pH adjustment | MEPS | • Sorbent: 80% C8 and 20% SCX<br>• Conditioning: 250 µL MeOH × 4 + 250 µL 0.1% formic acid × 4<br>• Sample loading cycles: 26<br>• Washing: 100 µL 3% acetic acid + 100 µL of 5 % MeOH<br>• Elution: 100 µL of 0.1% 10% $NH_4OH$ in MeOH (× 6<br>• Evaporation to dryness<br>• Derivatization | GC-MS | 0.1 | 53-78 | ≤ 14.25 | [205] |
| **THC-COOH** | Urine | 1 mL | • Alkaline hydrolysis<br>• pH adjustment | HF-LPME | • Extraction: 30 min (pH < 3, 10 mg NaCl, 1200 rpm)<br>• SLM: di-*n*-hexyl ether<br>• AP: NaOH 0.1 mM (30 µL)<br>• Evaporation to dyness<br>• Derivatization | GC-MS | 1.5 | 57.9-77.2 | ≤ 12.9 | [206] |
| **THC**<br>**CBN**<br>**CBD** | Saliva | 0.3 mL | • Dilution<br>• pH adjustment | DI-SPME | • Coating: PDMS<br>• Extraction: 30 min<br>• TD: 270ºC, 2 min | GC-MS | 0.5-2.0 | n.a. | ≤ 13.8 | [207] |
| **THC**<br>**CBN**<br>**CBD** | Breast milk | 0.05 mL | • Dilution<br>• pH adjustment | HS-SPME | • Sorbent: PDMS<br>• Extraction: 70ºC, 40 min, 25% NaCl<br>• TD: 250ºC | GC-MS | 10.0 | n.a. | ≤ 13.3 | [203] |
| **THC**<br>**11-OH-THC**<br>**THC-COOH**<br>**CBN**<br>**CBD**<br>**THCAA**<br>**CBG**<br>**THCV**<br>**THC-gluc**<br>**THC-COOH-gluc** | Urine | 0.2 mL | • Centrifugation<br>• Dilution<br>• Protein precipitation<br>• pH adjustment | DPX | • Sorbent: WAX<br>• Samples aspirated × 4<br>• Upper layer diluted and centrifuged | LC-MS/MS | 0.5-5.0 | 42.4-81.5 | ≤ 14.3 | [201] |
| **THC**<br>**11-OH-THC**<br>**THC-COOH**<br>**CBN**<br>**CBD**<br>**CBG**<br>**THCV**<br>**THCV-COOH** | Whole blood | 0.2 mL | • Protein precipitation<br>• Dilution<br>• Centrifugation<br>• pH adjustment | DPX | • Sorbent: WAX<br>• Samples aspirated 4 times<br>• Upper layer diluted and centrifuged | LC-MS/MS | 0.5-1.25 | 54.0-84.4 | ≤ 8.5 | [208] |

| | | | | | | | | | | |
|---|---|---|---|---|---|---|---|---|---|---|
| **THC-gluc**<br>**THC-COOH-gluc** | | | | | | | | | | |
| **THC**<br>**11-OH-THC**<br>**THC-COOH**<br>**CBN**<br>**CBD** | Urine | 0.09 mL | • Dilution<br>• pH adjustment<br>• Enzymatic and alkaline hydrolysis | DPX | • Sorbent: C18 (50 mg)<br>• Conditioning: 100 µL $H_2O$/ACN (1:1, v/v) × 2 + 100 µL $H_2O$/MeOH (60:40, v/v) × 3<br>• Samples loads/release cycles × 5 (200 µL)<br>• Washing: 100 µL $H_2O$ × 3<br>• Elution: 10 µL MeOH × 5 | LC-MS/MS | 2.0-4.0 | 65-85 | ≤ 15 | [209] |
| **THC**<br>**11-OH-THC**<br>**THC-COOH**<br>**CBN**<br>**CBD** | Saliva | 0.125 mL | • Dilution<br>• pH adjustment<br>• Protein precipitation<br>• Centrifugation | MEPS | • Sorbent: C18<br>• Conditioning: 250 µL MeOH (× 2) + 250 µL 1:1 $H_2O$/MeOH 50 mM COOH (× 2)<br>• Sample loading cycles: 5<br>• Washing: 100 µL 10mM COOH<br>• Elution: 25 µL of 50 mM $NH_4OH$ in MeOH (× 10) | LC-MS/MS | 0.008-0.12 | 50-105 | ≤ 14 | [210] |
| **THC**<br>**CBN**<br>**CBD** | Hair | 10 mg | • Washing<br>• Alkaline digestion<br>• Evaporation to dryness<br>• Dilution | HF-LPME | • Extraction: 20 min (pH 14, 6.8% NaCl, 600 rpm)<br>• SLM: butyl acetate<br>• AP: 20 µL butyl acetate | GC-MS | 0.5-15 pg/mg | 4.4-8.9 | ≤ 13.7 | [204] |
| **THC**<br>**CBN**<br>**CBD** | Hair | 10 mg | • Washing<br>• Alkaline digestion<br>• Evaporation to dryness<br>• Dilution | HS-SPME | • Sorbent: PDMS<br>• Extraction: 90ºC, 40 min, 1000 rpm<br>• TD: 250 ºC, 10 min | GC-MS | 0.007-0.062 | 1.1-8.7 | ≤ 16.4 | [211] |
| **11-OH-THC** | Urine | 1 mL | • Alkaline hydrolysis<br>• Dilution<br>• pH adjustment | SBSE | • Sorbent: PDMS<br>• Extraction: 120 min, 500 rpm, pH 3<br>• LD: ethyl acetate (1.5 mL), 30 min<br>• Evaporation to dryness | LC-MS/MS | 1.3 | 76.5 | ≤ 7.3 | [212] |
| **THC** | Saliva | 0.2 mL | • Centrifugation<br>• Dilution<br>• pH adjustment<br>• Filtration | PMME | • Sorbent: p(MAA-co-EGDMA)<br>• Conditioning: 0.3 mL MeOH + 0.3 mL phosphate buffer (20 mM, pH 7.0) at 0.15 mL/min<br>• Extraction: 1 mL at 0.08 mL/min<br>• Washing: 0.2 mL phosphate buffer (20 mM, pH 7.0) at 0.08 mL/min | GC-MS | 0.68 | 89 | ≤ 12 | [213] |

| | | | | | | | | | | |
|---|---|---|---|---|---|---|---|---|---|---|
| | | | | | • Elution: 0.05 mL acetone was at 0.04 mL/min | | | | | |
| **THC**<br>**11-OH-THC** | Blood | 1 mL | • Protein precipitation<br>• Dilution<br>• Centrifugation | DPX | • Sorbent: SDVB<br>• Conditioning: 800 µL $H_2O$/ACN (77:33, v/v)<br>• Samples loads/release cycles × 1<br>• Washing: 800 µL $H_2O$/ACN (77:33, v/v)<br>• Elution: 800 µL 50% ethyl acetate in ACN × 3<br>• Top layer evaporated to dryness<br>• Derivatization | GC-MS | 1-2 | 50-60 | ≤ 5.5 | [214] |
| **11-OH-THC** | Urine | 0.2 mL | • Alkaline hydrolysis<br>• Dilution<br>• pH adjustment | DPX | • Sorbent: SDVB<br>• Conditioning: 400 µL $H_2O$/ACN (77:33, v/v)<br>• Samples loads/release cycles × 1<br>• Washing: 400 µL $H_2O$/ACN (77:33, v/v)<br>• Elution: 400 µL 50 % ethyl acetate in ACN × 3<br>• Top layer evaporated to dryness<br>• Derivatization | GC-MS | 3 | n.a. | ≤ 4 | [214] |
| **THC**<br>**CBN**<br>**CBD** | Hair | 100 mg | • Washing<br>• Alkaline digestion<br>• Dilution<br>• Liquid-liquid extraction<br>• Evaporation<br>• Derivatization | HS-SPME | • Sorbent: PDMS<br>• Extraction: 125ºC, 20 min, 250 rpm<br>• TD: 270ºC, 10 min | GC-MS | 0.01-0.02 ng/mg | 14-66 | ≤ 13.4 | [215] |
| **THC**<br>**CBN**<br>**CBD** | Hair | 10 mg | • Washing<br>• Alkaline digestion<br>• Dilution<br>• pH adjustment | HS-SPME | • Sorbent: PDMS<br>• Extraction: 90ºC, 40 min<br>• TD: 250ºC, 20 min | GC-MS | 0.07 ng/mg | 36-92 | ≤ 18 | [216] |
| **THC**<br>**11-OH-THC**<br>**THC-COOH** | Plasma and urine | Urine: 1 mL<br><br>Plasma: 0.1 mL | • Dilution<br>• pH adjustment | µSPE | • Sorbent: MIP<br>• Conditioning: 5 0.1 M/0.1 M phosphate/NaOH buffer solution (pH 6.0) for 10 min<br>• Extraction: 150 rpm (12 min, 40ºC)<br>• Rinsing: 5 mL of 0.1 M/0.1 M phosphate/NaOH buffer solution at pH 6.0 for rinsing | HPLC-MS/MS | Urine: 0.14-0.16<br><br>Plasma: 0.11-0.15 | 87-94 | Urine: ≤ 6<br><br>Plasma: ≤ 11 | [202] |

| | | | | | | | | | |
|---|---|---|---|---|---|---|---|---|---|
| | | | | | (ultrasound assistance, 37 kHz, 325 W, 8 min)<br>• Elution: 2 mL of MeOH/aqueous acetic acid (90:10, v/v) through sonication (37 kHz, 325 W, 6 min) | | | | |
| **THC, CBN, 8THC, CBG, THCV, CBC, CBD** | Bucal swap | n.a. | • Headspace derivatization | HS-SPME | • Sorbent: PDMS<br>• Extraction: 150ºC, 5 min + 1 min<br>• TD: 0.5 min | GC-MS(SIM) | 0.2 µg/5 mg of buccal swab | n.a. | n.a. | [217] |
| **Anandamide, 2-arachidonoyl glycerol** | Plasma | 0.4 mL | • Protein precipitation<br>• Vacuum drying<br>• Redissolution<br>• pH adjustment | on-line in-tube SPME | • Sorbent: PIL<br>• Extraction: sampling flow rate of 0.1 mL/min, 2 min<br>• LD: 30:70 (v/v) mixture of 0.5% COOH in $H_2O$ and ACN with 0.5% COOH (0.1 mL/min, 2 min) | UHPLC-MS/MS | 0.05-0.1 (LLOQ) | n.a. | ≤ 14 | [218] |
| **THC** | Sweat | Cotton pad (0.5 × 0.5 cm) 4-6 rubbing on the forehead | • Alkaline extraction<br>• Salt addition<br>• pH adjustment | HS-SPME | • Coating: PDMS<br>• Extraction: 10 min (90ºC) + 30 min (150ºC) + 10 min (90ºC, for derivatization)<br>• TD: 3 min, 250ºC | GC-MS | 0.09 ng/pad | 100.7 | ≤ 9.3 | [89] |
| **THC**<br>**CBD**<br>**CBN** | Oral fluid | 1 mL | • Centrifugation | DI-SPME | • Coating: PDMS/DVB<br>• Extraction: 15 min<br>• TD: 270ºC (15 min | GC-MS | 1-5 | n.a. | ≤ 8.3 | [219] |
| **THC-COOH**<br>**CBD**<br>**OH-THC**<br>**THC**<br>**CBN** | Hair | 10 mg | • Washing<br>• Digestion<br>• PLE extraction<br>• Evaporation<br>• Redissolution<br>• Centrifugation<br>• Dilution<br>• pH adjustment<br>• Salt addition | PLE-DLLME | • DS: 500 µL of 2-propanol<br>• ES: 200 µL of chloroform<br>• Extraction: 24% NaCl, pH 11.0, 10% iso-propanol<br>• Sonication for 10 min<br>• Centrifugation (9000 rpm, 5 min, 3ºC)<br>• Infranatant collected, evaporated and redissolved | LC-HRMS/MS | 0.1-5 pg/mg | 73-87 | ≤ 25 | [100] |

**Table S5 – Dissociative Drugs**

| **Drugs** | Matrices | Sample amount | Sample pre-treatment | Microextraction technique | Optimized experimental conditions | Instrumental system | LOD (µg/L) | Absolute recovery (%) | Precision (%) | Ref |
|---|---|---|---|---|---|---|---|---|---|---|
| **PCP** | Urine<br>Whole blood | 1 mL | • Protein precipitation<br>• pH adjustment<br>• Salt addition | HS-SPME | • Coating: PDMS<br>• Extraction: 30 min, 50% $K_2CO_3$ (m/v) (900 rpm, 90ºC)<br>• TD: 250 ºC | GC-SID | 0.25-1.0 | 9.3-47.8 | ≤ 27 | [222] |
| **Dextromethorphan** | Urine<br>Plasma | Plasma: 0.8 mL<br>Urine: 2 mL | • Dilution<br>• pH adjustment | EME | • SLM: NPOE<br>• AP: 20 µL 0.1 M HCl + 15 µL 0.1 M NaOH<br>• Extraction: 20 min (110 V, 100 rpm) | DPV | 1.5 | 64-79 | ≤ 4.7 | [224] |
| **Dextromethorphan** | Urine<br>Plasma | 3 mL | • Filtration<br>• pH adjustment | HF-LPME | • SLM: *n*-C12<br>• AP: MeOH<br>• Extraction: 20 min (750 rpm) | IMS | 0.3-0.6 | 83-96 | ≤ 8 | [225] |
| **Salvinorin A** | Urine | 20 mL | • Protein precipitation<br>• pH adjustment<br>• Salt addition | DI-SPME | • Coating: PA<br>• Extraction: 30 min, (250 rpm, 30ºC)<br>• TD: 280ºC, 2 min | GCxGC-TOF/MS | 4 | n.a. | ≤ 6 | [226] |
| **Ketamine** | Urine | 3 mL | • pH adjustment<br>• Dilution<br>• Salt addition | SBSE | • Coating: titania-OH-TSO<br>• Extraction: 20 min (700 rpm, pH 11, 20% NaCl)<br>• LD: 60 µL 80/20 (v/v) MeOH/phosphate buffer,pH= 1.5 (20 min) | HPLC-UV | 9.1 | 90.8 | ≤ 8.9 | [194] |
| **Ketamine** | Urine | 0.5 mL | • pH adjustment<br>• Dilution<br>• Salt addition<br>• Filtration | DLLME | • DS: 350 µL ACN<br>• ES: 40 µL IL<br>• Hand-shaking (~ secs)<br>• Centrifugation (4000 rpm, 5 min)<br>• Supernatant collected<br>• Back-extraction with 60 µL 0.1 M HCl<br>• Hand-shaking (~ secs)<br>• Centrifugation (4000 rpm, 5 min) | CE-UV | 30 | n.a. | ≤ 12.8 | [227] |
| **PCP** | Urine | 2 mL | • Filtration<br>• pH adjustment | SDME | • Solvent: chloroform (2 µL)<br>• AP: (pH 10.5)<br>• Extraction: 8 min, 0.1 M NaOH | GC-PDHID | 70 | n.a. | ≤ 16.2 | [195] |
| **PCP**<br>**Ketamine** | hair | 10 mg | • Digestions | HS-SPME | • Coating: not specified | GC-MS | n.a. | 0.17-3.4 | ≤ 27 | [228] |

| | | | | | | | | | | |
|---|---|---|---|---|---|---|---|---|---|---|
| | | | • pH adjustment<br>• Salt addition | | • Extraction: 30 min, 8% $Na_2SO_4$ (m/v) (70ºC) + 15 min<br>• TD: 250ºC | | | | | |
| **Ketamine** | Urine Blood | 1 mL | • Dilution<br>• pH adjustment | HF-LPME | • SLM: toluene<br>• AP: toluene (10 µL)<br>• Extraction: 10 min (500 rpm, pH 13.0, 30ºC) | GC-MS | 2.5 | 81.3-98.6 | ≤ 4.5 | [92] |
| | | | | DLLME | • ES and DS: 100 µL toluene<br>• Sonication (3 min) and manual shaking<br>• Centrifugation (10000 rpm,3 min)<br>• Supernatant collected<br>• For blood samples, 10 mg of NaCl was added to break emulsion | | 1.5-2.5 | 87.3-103.4 | ≤ 3.5 | |
| **Ketamine norketamine (NK)** | Urine Plasma | 0.25 mL | • Centrifugation<br>• Dilution<br>• pH adjustment | MEPS | • Sorbent: 80% C8 and 20% SCX<br>• Conditioning: 5 × 250 µL MeOH + 4 × 250 µL $H_2O$<br>• Samples aspirated and discarded 8 or 26 times<br>• Washing: 250 µL of acetic acid<br>• Elution: 100 µL of 6% or 3 % $NH_4$ in MeOH<br>• Evaporation and redissolution<br>• Cleaning: 5 × 250 µL of MeOH + 4 × 100 µL of $H_2O$ | GC-MS/MS | 5 | 63-101 | ≤ 14 | [229] |
| **ketamine** | Urine | 3 mL | • pH adjustment<br>• Salt addition | HF-LPME | • SLM: o-xylene<br>• AP: o-xylene<br>• Extraction: 20 min (1000 rpm, pH 12.5, 30% NaCl, 30ºC) | GC-FID | 8 | n.a. | ≤ 8.9 | [174] |
| **Ketamine** | Sweat | Cotton pad (0.5 × 0.5 cm) 4-6 times rubbing on the forehead | • Acidic extraction<br>• pH adjustment | HS-SPME | • Coating: PDMS<br>• Extraction: 10 min (90ºC) + 3 min (90ºC) for derivatization<br>• TD: 3 min, 250 ºC | GC-MS | 0.07 ng/pad | 100.6 | ≤ 11.2 | [89] |
| **Ketamine** | Urine | 0.3 mL | Not necessary | DI-SPME | • Coating: C18/benzenesulfonic acid particle | MS | 0.027 | 90.8-109.4 | ≤ 10.6 | [230] |

| | | | | | | | | | | |
|---|---|---|---|---|---|---|---|---|---|---|
| | | | | | • Extraction: 10 min, (1500 rpm,)<br>• LD: 4 µL of 95:5 MeOH: $H_2O$ (0.1% COOH,12mM ammonium acetate), 3 min | | | | | |
| **Ketamine** | Whole Blood | 0.2 mL | • pH adjustment<br>• Dilution<br>• Salt addition | DLLME | • DS: 1.3 mL MeOH<br>• Vortex (2 min)<br>• Centrifugation (4000 rpm, 5 min)<br>• Supernatant collected<br>• ES: 0.2 mL DCM<br>• Sonication for 2 min<br>• Centrifugation (3500 rpm, 5 min)<br>• Infranatant injected | GC-MS | 10 | 86.3 | ≤ 11.1 | [145] |
| **Dextromethorphan and dextrorphan** | plasma | 0.5 mL | • Derivatization<br>• Dilution<br>• Salt addition<br>• pH adjustment | HS-SPME | • Coating: sol-gel PDMS<br>• Extraction: 30 min (70% of max rpm, pH 11.5-12, 20% NaCl, 60ºC)<br>• TD: 290ºC (5 min) | GC-MS | 0.010-0.015 | n.a. | ≤ 5 | [231] |
| **Ketamine** | Blood | 0.5 mL | • Protein precipitation<br>• Centrifugation<br>• Dilution<br>• Salt addition<br>• pH adjustment | DLLME | • DS: 0.25 mL of MeOH<br>• ES: 100 µL CHL<br>• Rapid injection<br>• Sonication (1 min)<br>• Centrifugation (4000 rpm,5 min)<br>• Infranatant collected, evaporated and redissolved | UPLC-MS/MS | 0.5 | 87-110 | ≤ 15 | [90] |
| **Ketamine Norketamine dehydronorketamine** | Urine | 2 mL | • Dilution<br>• Salt addition<br>• pH adjustment | HF-LPME | • SLM: eucalyptus essential oil<br>• AP: HCl 1.0 mol/L<br>• Extraction: 30 min (2400 rpm, pH > 10, 10% NaCl, 30 ºC)<br>• Evaporation to dryness<br>• Derivatization | GC-MS | 0.1-0.25 | 64.6-101.0 | ≤ 16.9 | [232] |
| **Ketamine** | Urine | 14 mL | • pH adjustment<br>• Dilution | HF-LPME | • SLM: *n*-C12<br>• AP: ACN (25 µL)<br>• Extraction: 30 min (700 rpm, pH 11.0, 20% NaCl) | GC-MS | 0.01 | 25-35 | ≤ 9.3 | [120] |

| | | | | | | | | | | |
|---|---|---|---|---|---|---|---|---|---|---|
| **Ketamine** | Hair | 20 mg | • Washing<br>• Drying<br>• Digestion<br>• pH adjustment<br>• Salt addition | HS-SPME | • Coating: PDMS<br>• Extraction: 5 min (90ºC, pH 10.3)<br>• TD: 250 ºC, 3 min | GC-MS | 0.59 ng/mg | n.a. | ≤ 17.52 | [98] |
| **Ketamine** | Urine | 1 mL | • Derivatization<br>• Dilution | HS-SPME | • Coating: PDMS<br>• Derivatization<br>• Extraction: 20 min (90ºC)<br>• TD: 225ºC, 1 min | GC-MS | 0.1 | n.a. | ≤ 14.8 | [196] |
| **Ketamine** | Urine | 1 mL | • Dilution<br>• Shaking<br>• Centrifugation<br>• Protein precipitation<br>• pH adjustment | MDSPE | • Coating: magnetic MWCNTs<br>• Extraction: 10 min (3000 rpm, 10% NaCl, pH 13)<br>• LD: 10 min sonication, 100 µL ethyl acetate | GC-MS | 0.024 | 80.1-93.6 | ≤ 9.2 | [157] |
| | Blood | | • Protein precipitation<br>• Shaking<br>• Centrifugation | | | | | | | |
| **PCP** | Urine | 4 mL | • Dilution<br>• Salt addition<br>• pH adjustment | DLLME | • DS: 1505 µL ACN<br>• ES: 606 µL $CH_2Br_2$<br>• Extraction: 30%-$NH_3$ pH ≥ 11.5<br>• Rapid injection<br>• Centrifugation (9500 rpm,5 min)<br>• Infranatant collected, evaporated and redissolved | CE-UV | 2.9-5.6 | 80.3 | ≤ 11.0 | [164] |
| **PCP**<br>**Ketamine** | Oral fluid | 90 µL | • Centrifugation<br>• pH adjustment<br>• Dilution | µSPE | • Sorbent: C18<br>• Extraction: 15 min (30ºC, 200 rpm)<br>• Washing: 100 µL $H_2O$<br>• Elution: 100 µL with MeOH containing 10 mM of COOH | LC-MS/MS | 0.1-0.5 | 79-85 | ≤ 8 | [129] |
| **PCP**<br>**Ketamine** | Urine<br>Plasma | Urine: 90 µL<br>Plasma: 180 µL | • Urine samples were diluted<br>• Sonication<br>• Centrifugation | µSPE | • Sorbent: C18<br>• Washing: 100 µL $H_2O$<br>• Elution: 100 µL with MeOH containing 10 mM of COOH | LC-MS/MS | 0.1-0.3 | 80-90 | ≤ 6 | [130] |

| | | | | | | | | | | |
|---|---|---|---|---|---|---|---|---|---|---|
| | | | • pH adjustment<br>• Dilution | | | | | | | |
| **Ketamine** | Oral fluid | 0.025 mL | • Dilution<br>• Centrifugation<br>• pH adjustment | MEPS | • Sorbent: C18<br>• Conditioning: 100 μL MeOH + 100 μL $H_2O$<br>• Samples aspirated × 5 (50 μL)<br>• Elution: 25×50 DCM:2-propanol:$NH_4OH$<br>• Cleaning: 10×50 μL | DESI-HRMS | 0.5 mg/L (LLOQ) | n.a. | ≤ 9.6 | [233] |
| **Ketamine** | Blood | 0.5 mL | • Protein precipitation<br>• Centrifugation<br>• Dilution<br>• Salt addition<br>• pH adjustment | DLLME | • DS + ES: 350 μL of a mixture chloroform/MeOH 1:2.5,<br>• Rapid injection<br>• Sonication (2 min)<br>• Centrifugation (4000 rpm,5 min)<br>• Infranatant collected, evaporated and redissolved | UPLC-MS/MS | 0.5 | 51-75 | n.a. | [234] |
| **Ketamine**<br>**PCP** | Hair | 10 mg | • Washing<br>• Digestion<br>• PLE extraction<br>• Evaporation<br>• Redissolution<br>• Centrifugation<br>• Dilution<br>• pH adjustment<br>• Salt addition | PLE-DLLME | • DS: 500 μL of 2-propanol<br>• ES: 200 μL of chloroform<br>• Extraction: 24% NaCl, pH 11.0, 10% iso-propanol<br>• Ultrasound for 10 min<br>• Centrifugation (9000 rpm, 5 min, 3ºC)<br>• Infranatant collected, evaporated and redissolved | HPLC-HRMS/MS | 1-2 pg/mg | 48-60 | ≤ 11 | [100] |
| **Ketamine**<br>**Norketamine** | Urine | 0.5 mL | • Centrifugation<br>• Acid hydrolysis<br>• pH adjustment<br>• Dilution | HT-BAμE | • Sorbent:NVP-DVB<br>• Extraction: 30 min (1800 rpm) pH 11.0<br>• LD: sonication with 100 μL MeOH (15 min) | GC-MS | 1.0 | 84.9-105.0 | ≤ 12.6 | [223] |
| **Ketamine** | Urine | 1.5 mL | • Dilution<br>• pH adjustment | EME | • Extraction: 20 min (pH 4, 180 V) 1000 rpm<br>• SLM: Porous Aromatic Framework-48 with the nitro functional groups with NPOE | IMS | 2.7 | 92-97 | ≤ 4.3 | [105] |

| |
|---|
| • AP: 100 mmol/L HCl (20 µL) |

**Table S6 - NPS**

| **Drugs** | Matrices | Sample amount | Sample pre-treatment | Microextraction technique | Optimized experimental conditions | Instrumental system | LOD (µg/L) | Absolute Recovery (%) | Precision (%) | Ref |
|---|---|---|---|---|---|---|---|---|---|---|
| **Synthetic cathinones**<br>MPD, BFD, 4-MEPE and PTL | Urine | 1 mL | • pH adjustment<br>• Salt addition | DI-SPME | • Coating: PDMS/DVB<br>• Extraction: 60 min, 42 % NaCl (m/v), pH 12.6 (2000 rpm)<br>• LD: 65 µL MeOH + 10 µL HCl:MeOH (1:9, v:v) (2000 rpm)<br>• Evaporation and derivatization<br>• Evaporation and redissolution | GC-MS | 5-25 | 2-80 | ≤ 15 | [197] |
| **Synthetic cannabinoids**<br>UR-144, JWH-250, CP47497, CP47497-C8, JWH-200, JWH-081, AM 2201, JWH-019, JWH-122 | Oral fluid | 1 mL | • Centrifugation | DI-SPME | • Coating: PDMS/DVB<br>• Extraction: 15 min<br>• TD: 270ºC (15 min) | GC-MS | 1-10 | n.a. | ≤ 12.8 | [219] |
| **Synthetic cathinones**<br>MTL, FPD, ECAT ECATEPE, EL, MEPE, BL, MPD, PD, MDPV, PV<br><br>**Synthetic opioid**<br>EDDP<br>PBN | Oral fluid | 0.06 mL | • Dilution<br>• Centrifugation<br>• pH adjustment | MEPS | • Sorbent: C8/SCX<br>• Conditioning: 100 µL MeOH + 100 µL $H_2O$<br>• Samples aspirated and discarded × 6<br>• Washing: 50 µL of $H_2O$/MeOH 90:10 (v:v)<br>• Drying (0.5 min)<br>• Elution: 90 µL of DCM/IPA/$NH_4OH$ (78:20:2, v:v:v)<br>• Evaporation and redissolution<br>• Cleaning: 100 µL of eluent, 100 µL of MeOH, 100 µL of $H_2O$ (× 4), 100 µL 0.1% COOH (× 4) and 100 µL MeOH (× 4) | UPLC-MS/MS | 0.25 | 42-145 | < 8.9 | [128] |
| **Synthetic cathinones**<br>MPD<br><br>**Synthetic cannabinoid**<br>UR-144, JWH-250, JWH-200, JWH-122, JWH- 019, AM-2201, JWH-081, HU-211, CP47497 | Oral fluid | 0.025 mL | • Dilution<br>• Centrifugation<br>• pH adjustment | MEPS | • Sorbent: C18<br>• Conditioning: 100 µL MeOH + 100 µL $H_2O$<br>• Samples aspirated × 5 (50 µL)<br>• Elution: 50 µL DCM/IPA/$NH_4OH$ × 25<br>• Cleaning: 50 µL x 10 | DESI-HRMS | 0.25-0.5 mg/L (LLOQ) | n.a. | < 19.4 | [233] |
| **Synthetic cathinones** | Oral fluid | 0.5 mL | • Dilution<br>• Salt addition | US-DLLME | • DS: 1.4 mL MeOH<br>• ES: 200 µL $CHCl_3$ | UPLC-MS/MS | 0.1-2.5 | 74.6-125.3 | ≤ 14.8 | [99] |

| | | | | | | | | | | |
|---|---|---|---|---|---|---|---|---|---|---|
| ML, FPD, ECAT, ECATEPE, EL, MEPE, BL, MPD, PD, MDPV, PV<br><br>**Synthetic opioid**<br>EDDP, BPN | | | • Protein precipitation | | • Extraction: 2.5 % NaCl (m/v), pH 8.0<br>• Ultrasound for 5 min<br>• Centrifugation (3500 rpm, 5 min)<br>• Infranatant collected, evaporated and redissolved | | | | | |
| **Synthetic cathinones**<br>4-FMC, CAT, NM2AI, 3-MMC, PD, MBDB, 4-MTA, ML, EL, BL, PL, α-PVP, 2-CB, MET, 4-MEC, MDPV, 4-MMC | Urine | 2 mL | • Dilution<br>• pH adjustment<br>• NaCl addition<br>• Derivatization | DLLME | • DS: 350 μL 1:2.5 $CHCl_3$/MeOH (v:v) through rapid injection<br>• Centrifugation (4400 rpm, 4 min)<br>• Infranatant injected | GC-MS | 2-50 | 92-115 | ≤ 14.8 | [143] |
| | Blood | | • Protein precipitation<br>• Centrifugation<br>• Dilution<br>• pH adjustment<br>• NaCl addition<br>• Derivatization | | | | | | | |
| **Synthetic cannabinoids** JWH-200, AB-005, JWH-018 5OH pentyl, AM-2201 4OH pentyl, JWH-018 4OH pentyl, UR-144 4OH pentyl, MAM-2201, JWH-250, JWH-073, XLR-11, JWH-018, | Plasma | 0.1 mL | • Dilution<br>• Protein precipitation<br>• Centrifugation | μSPE | • Sorbent: C18<br>• Extraction: load and eject x 5<br>• Washing: 100 μL MeOH:$H_2O$ (80:20, v:v)<br>• Elution: 100 μL 10 mM COOH in MeOH × 3 | LC-MS/MS | 0.1-3.0 | 21-70 | ≤ 25 | [238] |

| | | | | | | | | | | |
|---|---|---|---|---|---|---|---|---|---|---|
| JWH-081, JWH- 122, UR-144<br><br>**Synthetic cathinones**<br>MPD, 4-MEC, MDPV<br>*α*-PVP, 2C-B, 2C-T-7 | | | | | | | | | | |
| **Piperaine derivatives**<br>BZP, TFMPP, mCPP, MeOPP | Urine | 0.1 mL | • Dilution | MEPS | • Sorbent: 80 % C8 and 20 % SCX<br>• Conditioning: 100 μL MeOH + 100 μL $H_2O$<br>• Samples aspirated × 8<br>• Washing: 250 μL 1% acetic acid and 100 μL 10 % MeOH in $H_2O$ (v:v)<br>• Elution: 50 μL 5 % $NH_3$ in MeOH (v:v)<br>• Cleaning: 5 × 250 μL MeOH and 4 × 250 μL $H_2O$ | HPLC-DAD | 50-100 | 52.0-100.5 | ≤ 16.6 | [239] |
| **Piperaine derivatives**<br>BZP, TFMPP, mCPP, MeOPP | Urine | 0.1 mL | • Dilution | MEPS | • Sorbent: C18<br>• Conditioning: 100 μL MeOH + 100 μL $H_2O$<br>• Samples aspirated × 8<br>• Washing: 250 μL of 10 % MeOH in $H_2O$ (v:v)<br>• Elution: 50 μL of MeOH<br>• Cleaning: 5 × 250 μL MeOH and 4 × 250 μL $H_2O$ | HPLC-DAD | 50-100 | 52.0-100.5 | ≤ 16.6 | [240] |
| **Mitragynine** | Urine | 1 mL | • Dilution | BAμE | • Sorbent: NVP<br>• Extraction: 4 h (1300 rpm), pH 5.5<br>• Elution: 200 μL MeOH/ ACN (1:1, v:v) under sonication (10 min) | HPLC-DAD | 0.1 | 103 | ≤ 15 | [237] |

| | | | | | | | | | | |
|---|---|---|---|---|---|---|---|---|---|---|
| **Synthetic cannabinoids**<br>AM-2201, AM-2233, AM-694, CB-13, JWH-007, JWH-019, JWH-015, JWH-018, JWH-030, JWH-073, JWH-081, JWH-098, JWH-122, JWH-147, JWH-200, JWH-201, JWH-250, JWH-251, JWH-307, JWH-398, RCS4, JWH-018 4OH indole, JWH- 018 5OH pentyl, JWH-018-COOH, JWH-073 4OH butyl, JWH-073 5OH indole, JWH-073 COOH, JWH-250 5OH pentyl<br><br>**Synthetic cathinones**<br>4-FAMP, 4-MEC, BL, BPD, CAT, EL, EPN, HML, HMO, MBDB, MDAI, MDPV, MPD, MD, ML, 4- MTA, NM-2-AI, PD, PL<br><br>**Piperaine derivatives**<br>BZP, mCPP | Blood | 0.5 mL | • Protein precipitation<br>• Centrifugation<br>• Dilution<br>• Salt addition<br>• pH adjustment | DLLME | • DS + ES: 350 μL of $CHCl_3$/MeOH 1:2.5 (v:v),<br>• Rapid injection<br>• Sonication (2 min)<br>• Centrifugation (4000 rpm,5 min)<br>• Infranatant collected, evaporated and redissolved | UHPLC-MS/MS | 0.2 | 4-110 | n.a. | [234] |
| **MDPV** | Oral fluid | 0.5 mL | Not necessary | LLME | • ES: 100 μL $CHCl_3$<br>• Centrifugation 3500 rpm (3 min) | IMS | 4.4 | n.a. | ≤ 3.2 | [241] |
| **Synthetic cannabinoids** AM-1220, JWH-200, AB-005, JWH-018-COOH JWH-018 5OH pentyl, WIN-55, | Oral fluid | 0.09 mL | • Dilution<br>• Centrifugation<br>• Protein precipitation<br>• Salt addition<br>• pH adjustment | MEPS | • Sorbent: C18<br>• Conditioning: 250 μL $H_2O$/MeOH (75:25, v:v) × 3<br>• Samples aspirated × 5<br>• Washing: 200 μL $H_2O$/MeOH (90:10, v:v) × 3 | UHPLC-MS/MS | 0.005-0.850 | 31-96 | ≤ 20 | [242] |

| | | | | | | | | | | |
|---|---|---|---|---|---|---|---|---|---|---|
| XLR-11 4OH pentyl, MAM-2201 COOH, JWH-073, UR-144 4OH pentyl, JWH-250, MAM-2201, XLR-11, JWH-018, JWH-081, JWH-122, UR-144<br><br>**Synthetic cathinones**<br>ML,DMCAT, MPD, ECAT BPD, BL, MD, 4-MEC, MET, MDPV, α-PVP, 2-CB<br><br>**Piperazine derivatives**<br>PPP, MeOPP | | | | | • Elution: 100 µL MeOH containing 10 mM COOH × 5<br>• Dilution with 100 µL of water | | | | | |
| **Synthetic cathinones**<br>ML, BL, CAT, EL, MDPV, PL, PD, NPR, FPD, 3,4-DMMC | Urine | 1 mL | • pH adjustment | µSPE | • Sorbent: MIP<br>• Extraction: 200 rpm, 4 min<br>• Washing: 2 mL 0.1 M phosphate buffer, pH 5.0 (orbital/horizontal shaking of 100 rpm for 10 min)<br>• Elution: ultrasound (37 kHz) treatment with 2 mL of 75:20:5 *n*-C7/IPA/ $NH_4OH$ (v:v:v) for 4 min<br>• Evaporation to dryness and redissolution | HPLC-MS/MS | 0.14-1.51 | 89-100 | ≤ 10 | [243] |
| **Synthetic cathinones**<br>2-FAMP, 3-FAMP, 2-FMAMP, 4-FMAMP, 3-FEAMP, 3-MMAMP, 6-APB, ETCAT, 4-CECAT, 4-CMCAT, 4-MeMABP, α-PVP, α-PHP, MPHP, MXD, ML, BL, MDPPP, MDPBP, 2C-E | Oral fluid | 0.2 mL | • Dilution<br>• pH adjustment | DPX | • Sorbent: MP<br>• Conditioning: 200 µL 1 % acetic acid in MeOH and 200 µL $H_2O$<br>• Samples aspirated and dispensed × 10<br>• Washing: 200 µL 10% IPA in $H_2O$ (v:v) and dried twice with 200 µL air<br>• Elution: 100 µL 1 % acetic acid in MeOH (v:v) × 5 | UHPLC-MS/MS | 0.1 | 64-115 | ≤ 8.4 | [131] |
| **DCP** | Oral fluid | 0.09 mL | • Centrifugation<br>• pH adjustment | MEPS | • Sorbent: C8<br>• Conditioning: 3 × 100 µL IPA and 2 × 100 µL $H_2O$<br>• Extraction: 100 µL sample (adjusted to pH 7 × 4 | IMS | 30 | Not available | ≤ 14 | [244] |

| | | | | | | | | | |
|---|---|---|---|---|---|---|---|---|---|
| | | | | | • Washing: 100 µL $H_2O$ Elution: 50 µL IPA × 10<br>• Dilution with 100 µL $H_2O$ | | | | | |
| **Synthetic cathinones** MDAI, ML, 4-FAMP, mCPP, PD, MET, MDPV, EPN, 2C-E, bromo-dragonfly, and AH-7921 | Plasma Whole blood | Plasma: 0.125 mL<br><br>Blood: 0.1 mL | • Dilution<br>• pH adjustment | PALME | • SLM: 5 µL DCA with 1 % TOA (w/w)<br>• AP: 50 µL 20 mM COOH<br>• Extraction: alkaline solution (900 rpm, 120 min) | UHPLC–MS | 0.1-147 (LLOQ) | 11-117 | ≤ 20 | [245] |
| **Synthetic cathinones** 2-FMC, ML, BPD, 2C-H, 2-MMC, BL, MPD, DECAT, 4-MEC, MET, 2-CB, α-PVP, MDPV, NBPN, 2C-T-4, 2C-T-7<br><br>**Synthetic cannabinoid** BPN, JWH-200, EDDP, XLR-11 OH pentyl, JWH-018-COOH, WIN- 55, JWH-018 5OH pentyl, JWH-018 4OH pentyl, MAM-2201 COOH, UR-144 4OH pentyl, JWH-081 5OH pentyl, AM-1220, 25I-NBOMe, AB-005, JWH-250, MT-45, JWH-073, XLR-11, MAM-2201, JWH-018, JWH-081, UR144, JWH-122 | Hair | 10 mg | • Washing<br>• Digestion<br>• PLE extraction<br>• Evaporation<br>• Redissolution<br>• Centrifugation<br>• Dilution<br>• pH adjustment<br>• Salt addition | PLE-DLLME | • DS: 500 µL of IPA<br>• ES: 200 µL of $CHCl_3$<br>• Extraction: 24 % NaCl, pH 11.0, 10% IPA<br>• Ultrassound for 10 min<br>• Centrifugation (9000 rpm, 5 min, 3ºC)<br>• Infranatant collected, evaporated and redissolved | HPLC-HRMS/MS | 0.1-5 pg/mg | 23-87 | ≤ 25 | [100] |
| **Synthetic cannabinoid** CP47497, JWH-030, JWH-251, JWH-203, JWH-302, JWH-201, RCS4, JWH-250, JWH-015, JWH-075, | Urine and blood | 2 mL | • Hydrolysis<br>• pH adjustment<br>• Salt addition<br>• Protein precipitation | US-DLLME | • ES: 150 µL of $CHCl_3$, rapid injection<br>• Sonication (5 min)<br>• Centrifugation (4400 rpm, 5 min)<br>• Infranatant collected, evaporated, derivatized and redissolved | GC-MS | 1-5 | 47.8-100.0 | ≤ 15.8 | [246] |

| |
|---|
| AM-694, JWH-018, JWH-307, JWH-019, AM-2201, JWH-122, JWH-210, JWH-147, JWH-081, JWH-098, JWH-200, UR-144, XLR-11, AB-PINACA, AB-CHMINACA, AB-FUBINACA, AKB-48, JWH-007, JWH-250-5OH, JWH-018-4OH, JWH-018-5OH, JWH-018-COOH, JWH-073-4OH, JWH-073-5OH, JWH-073-COOH, |
